# Supplementary material for: Chimeric systems composed of swapped Tra subunits between distantly-related F plasmids reveal striking plasticity among type IV secretion machines
Source: PLoS Genet. 2024 Mar 4;20(3):e1011088. doi: 10.1371/journal.pgen.1011088 (PMC10939261; doi:10.1371/journal.pgen.1011088)

A

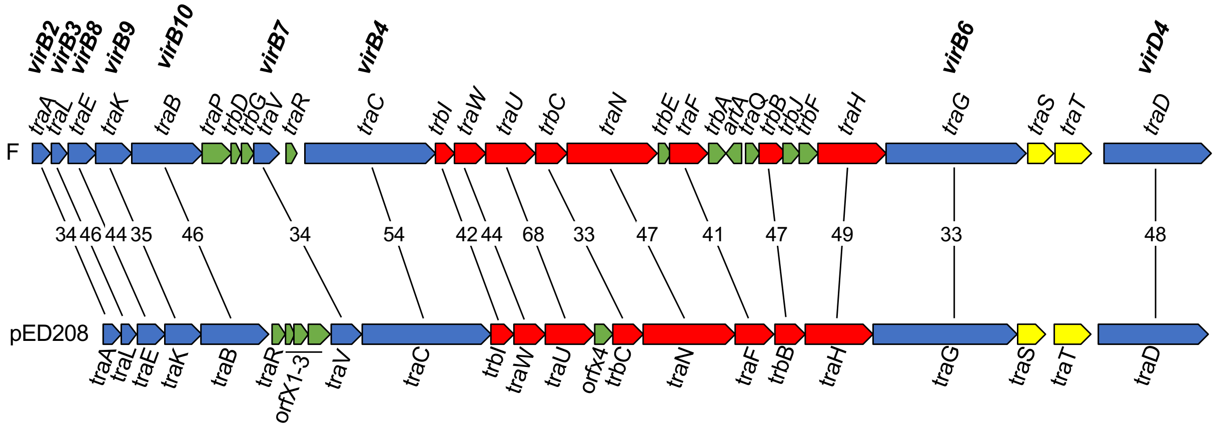

S1 Fig. Alignments of Tra/Trb protein homologs. Panel A: Alignment of the F and pED208 *tra/trb* regions. **Blue:** VirB/VirD4-like scaffold subunits that are conserved among T4SSs. **Red:** F-specific proteins that contribute to assembly or function of F-like T4SSs. **Yellow:** F-specific proteins involved in surface exclusion. **Green:** Nonconserved proteins. Lines connect genes encoding protein homologs; numbers correspond to the percent amino acid identities across the lengths of the homologs. Accession numbers: pED208 (NCBI Bioproject PRJNA871772); F (NZ_MF370216). By comparison, most F/pED208 VirB/VirD4 subunits exhibit ~18-20 % sequence identities with their counterparts in the 'minimized' pKM101-encoded system, except for the VirB6-like TraG$_{F}$/TraD$_{KM}$ (8% identical) and VirB7-like TraV$_{F}$/TraN$_{KM}$ (~6% identical) homologs.

Panels B - F (following pages): Similarities among the Tra/Trb homologs at the primary sequence and structural levels, grouped as indicated (orange highlights). Upper: Sequence alignments of the Tra/Trb proteins indicated, generated with Multalin [85]. Known domains and percent identities are shown for regions indicated. Red lines denote regions of sequence divergence, one or more of which might confer the distinct phenotypes accompanying subunit swapping described in the text. Lower: Structures of the Tra/Trb homologs predicted from the AlphaFold Protein Structure Database [82]. The solved TraA and TraB$_{ED}$ structures were from RCSB PDB. The structure of pKM101-encoded TraJ was predicted by ColabFold [83]. Structures of the homologs are presented separately and superimposed. For TraB, only the β-barrel and AP domains comprising the OMCC and AP channel are shown. Percent identities of the structurally aligned sequences, Root Mean Square Deviation (RMSD), TM-score, and number of equivalent residues relative to the entire sequence lengths were derived from the RCSB PDB Pairwise Structure Alignment website (https://www.rcsb.org/alignment).

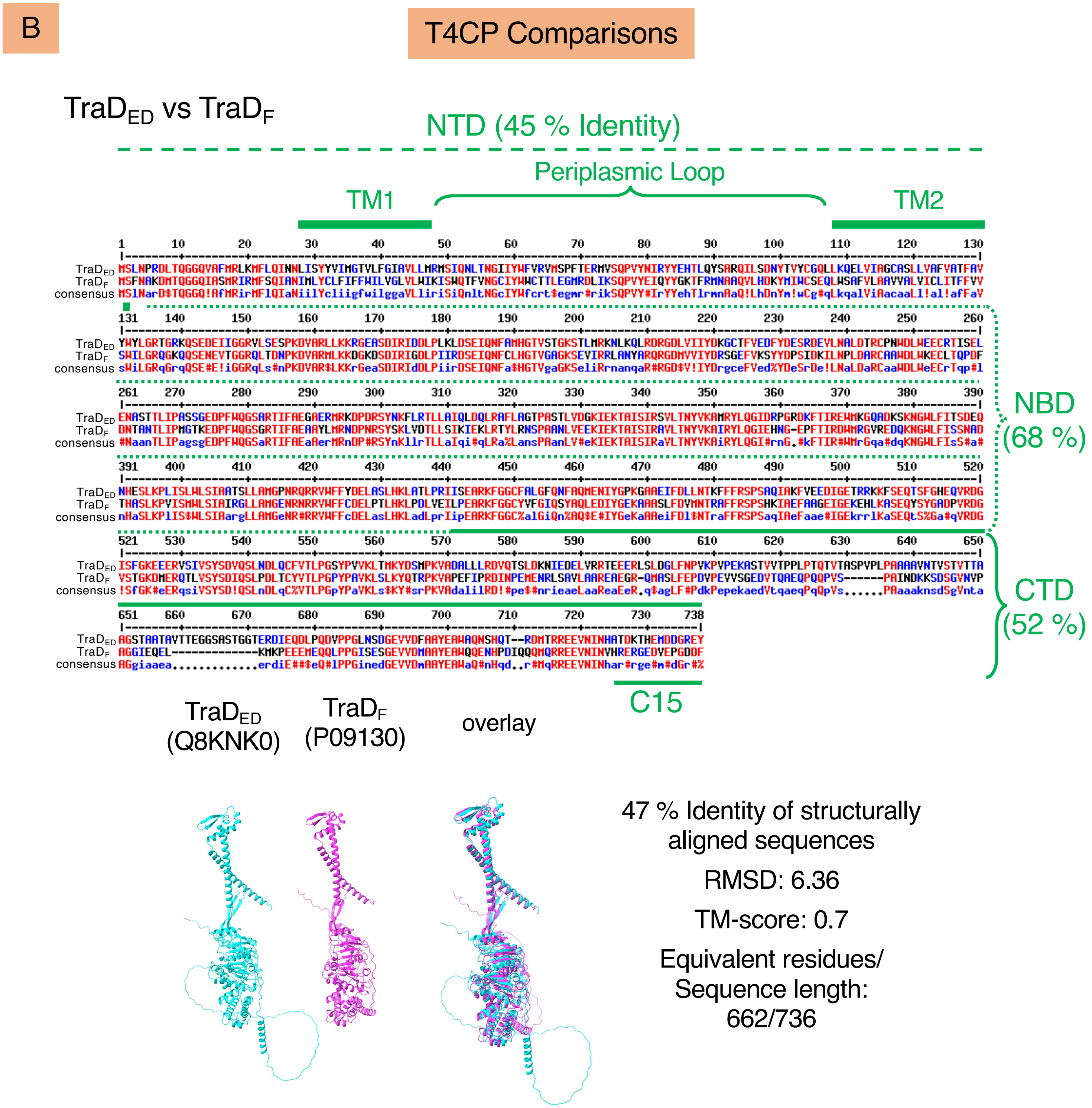
B
T4CP Comparisons
TraDED vs TraDF
NTD (45 % Identity)
Periplasmic Loop
TM1
TM2
NBD (68 %)
CTD (52 %)
C15
TraDED (Q8KNK0)
TraDF (P09130)
overlay
47 % Identity of structurally aligned sequences
RMSD: 6.36
TM-score: 0.7
Equivalent residues/ Sequence length: 662/736

$TraD_{ED}$ vs $TraJ_{KM}$

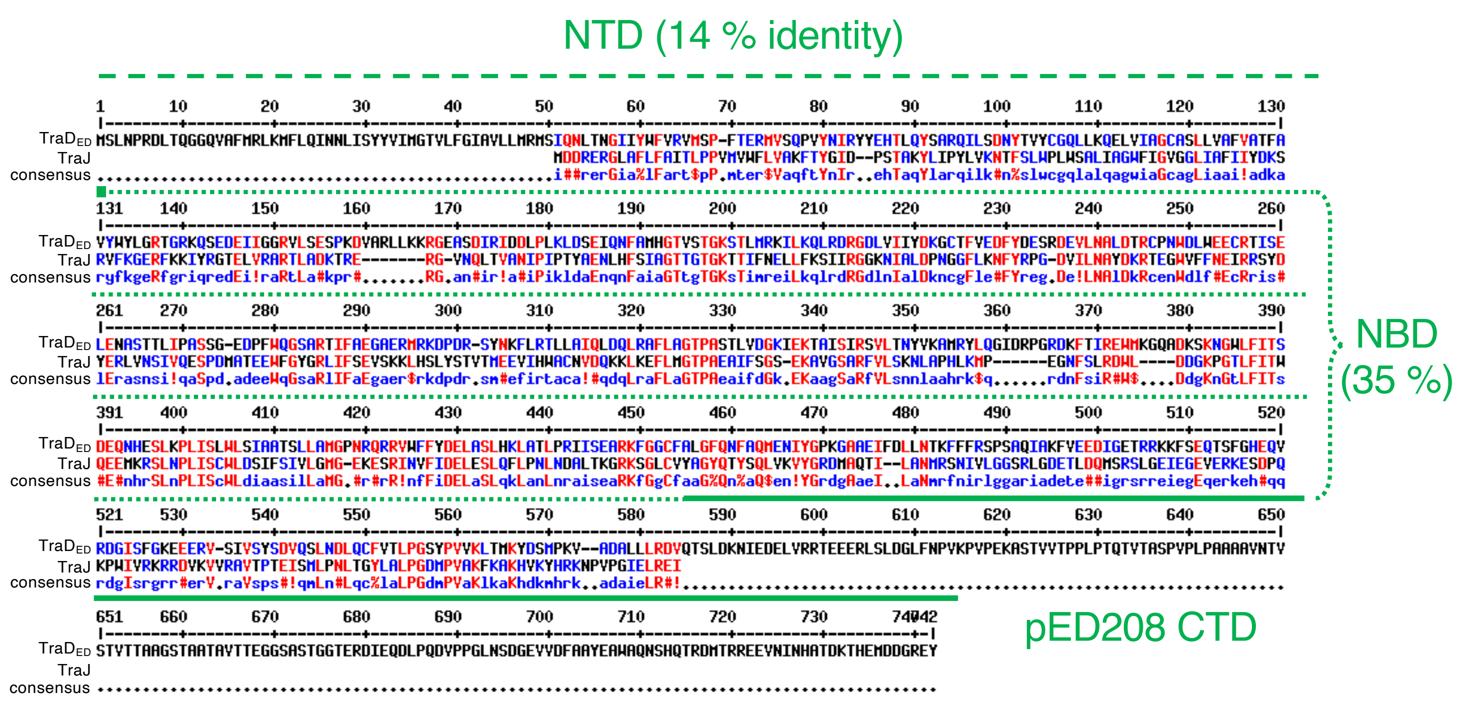

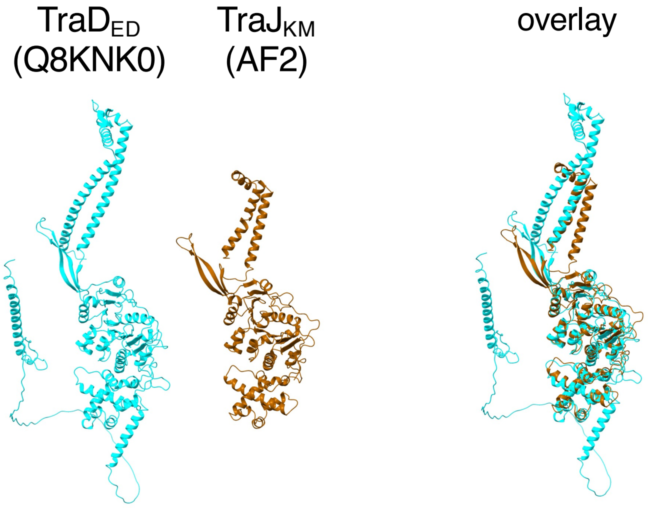

23 % identity of structurally aligned sequences

RMSD: 4.81

TM-score: 0.55

Equivalent residues/ Sequence length: 509/736

C

IMC Subunit Comparisons

TraL

```
                1       10        20        30        40        50        60        70        80        90        100103
                |--------+---------+---------+---------+---------+---------+---------+---------+---------+---------+--|
TraLED           MEGNDLDKYRFPKTLSEQNRIIGLPLDEAIPAAYVLLWGFFTKKYLFSLIIAAYIWQLIRAAKRGKSSRWLYNWCYWYLPTELFRVYYRVIPDSSFRKWIK
TraLF           MSGDENKLKKYRFPETLTNQSRWFGLPLDELIPAAICIGWGITTSKYLFGIGAAVLVYFGIKKLKKGRGSSWLRDLIYWYMPTALLRGIFHNVPDSCFRQWIK
consensus       ..g#eNdLdKYRFPeTLs#QnRiiGLPLDEaIPAA!cigWGifTkKYLFgigaAal!wqgIraaKrGrgSrWLr#lcYWY$PTaLlRg!%rn!PDScFRqWIK
```

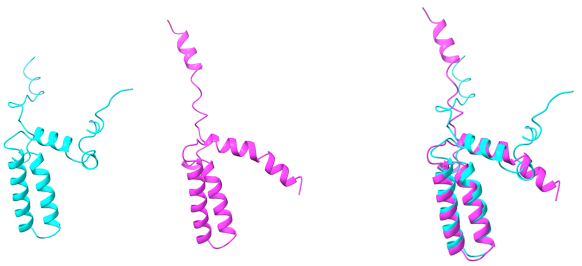

49 % Identity

RMSD: 3.65

TM-score: 0.55

Equivalent residues/
Sequence length:
78/101

TraC

```
           1       10        20        30        40        50        60        70        80        90        100       110       120       130
           |--------+---------+---------+---------+---------+---------+---------+---------+---------+---------+---------+---------+---------|
TraCED         MKKFLNVLENLQNAFRMPDGASEMTRNLASLDFPQLTSVLPYRDYDRDSGLFVNRSTVGFLLRAEPLIGANEQIVYVLEDLIKSKLPRKTPISFHLVSSKLIGDQLDRGLSDFRWQGKRADKFNRI
TraCF      MNNPLEAVTQAVNSLVTALKLPDESAKANEVLGEMSFPQFSRLLPYRDYNQESGLFMNDTTMGFMLEAIPINGANESIVEALDHMLRTKLPRGIPLCIHLMSSQLVGDRIEYGLREFSWSGEQAERFNAI
consensus  ....$eafl#al#nLqnAlr$PDeaaeanrnLae$dFPQlsrlLPYRDY#r#SGLFmNrsTmGF$LrAePinGANEqIVeaL#d$irsKLPRgiPiciHLmSSqL!GDri#rGLr#FrWqGerA#rFNaI

           131     140       150       160       170       180       190       200       210       220       230       240       250       260
           |--------+---------+---------+---------+---------+---------+---------+---------+---------+---------+---------+---------+---------|
TraCED     TRAFYQRAAQQQFNSPTG--LPLTLRDYRLYISYCVKAKKRTAAVMTEVAHTLKVLRSSLDAAKIYTQAADDQELATLVHGMLNARHDQLYPDEVRVGKFDELHQRCVDQRIGIDVRPDDVLISLADRQG
TraCF      TRAYYMKAAATQFPLPEGMNLPLTLRHYRVFISYCSPSKKKSRADILEMENLVKIIRASLQGASITTQTVDAQAFIDIVGEMINHNPDSLYPKRRQLDPYSDLNYQCVEDSFDLKVRADYLTLGLREN-G
consensus  TRA%YqrAAaqQFnlPeG..LPLTLRdYRl%ISYCskaKKrsaAdilEmanllK!iRaSL#aAkItTQaaDaQaladiVgeMiNarhDqLYPdrrrldk%d#LnqrCV##rididVRaDdlligLa#r.G

           261     270       280       290       300       310       320       330       340       350       360       370       380       390
           |--------+---------+---------+---------+---------+---------+---------+---------+---------+---------+---------+---------+---------|
TraCED     SVTSTRAMNFMLENNPDMFMLWMGGDNLSNLLSPDLTIASPFVITMVMEAEDQVATQGEATRKYLDRDKKANSPYATLFPGVAKQAKEWKEIREHLNSNQTCIVRYYYNVTTFCPDTDEDALVCEQQVIN
TraCF      RNSTARILNFHLARNPEIAFLWNMADNYSNLLNPELSISCPFILTLTLVVEDQVKTHSEANLKYMDLEKKSKTSYAKWFPSVEKEAKEWGELRQRLGSGQSSVVSYFLNITAFCKDNNETALEVEQDILN
consensus  rnssaRa$NFhLarNP#iamLWngaDNlSNLLnP#LsIacPF!iT$t$eaEDQVaTqgEAnrKY$Dr#KKanspYAklFPgVaK#AKEWgEiR#rLnSnQsc!VrY%lN!TaFCkDn#EdALecEQ#!iN

           391     400       410       420       430       440       450       460       470       480       490       500       510       520
           |--------+---------+---------+---------+---------+---------+---------+---------+---------+---------+---------+---------+---------|
TraCED     TFKKNGIDLFSPTYMQFRNWIAMIPFIHAEGLWEDIKMGGATCRAESIQAVNLLPVVADNRLCQGGLLTPSYRNQLAFLDIYGDGMGNTNYNMAVTGTSGAGKTGLVQPILRSVLDSGGIAWVFDMGDGY
TraCF      SFRKNGFELISPRFNHMRNFLTCLPFMAGKGLFKQLKEAGVVQRAESFNVANLMPLVADNPLTPAGLLAPTYRNQLAFIDIFFRGMNNTNYNMAVCGTSGAGKTGLIQPLIRSVLDSGGFAVVFDMGDGY
consensus  sFrKNGi#LiSPr%nqmRNfiaciPFiaaeGLfe#iKeaGatcRAESi#aaNL$PlVADNrLcqaGLLaPsYRNQLAFiDI%grGMnNTNYNMAVcGTSGAGKTGL!QPiiRSVLDSGGiAwVFDMGDGY

           521     530       540       550       560       570       580       590       600       610       620       630       640       650
           |--------+---------+---------+---------+---------+---------+---------+---------+---------+---------+---------+---------+---------|
TraCED     KSFCENVGGTYLDGKSLKFNPFANISSINESGERIRDQLAVLASPNGTLDEVHHSLLLRGVQEAWEAHREKARIDHVVQKLTAIREDDKYQNSPGITNRLDEIIELLGKYCSWGIYGEYFNSDEPSLTDD
TraCF      KSLCENMGGVYLDGETLRFNPFANITDIDQSAERVRDQLSVMASPNGNLDEVHEGLLLQAVRASWLAKENRARIDDVVDFLKNASDSEQYAESPTIRSRLDEMIVLLDQYTANGTYGQYFNSDEPSLRDD
consensus  KSlCENmGGtYLDGesLrFNPFANIsdI##SaER!RDQLaV$ASPNGnLDEVHegLLLraVraaWeAhr#rARIDdVV#kLkaar#d#qYa#SPgIrnRLDEiIeLLdqYcanGiYG#YFNSDEPSLrDD

           651     660       670       680       690       700       710       720       730       740       750       760       770       780
           |--------+---------+---------+---------+---------+---------+---------+---------+---------+---------+---------+---------+---------|
TraCED     ARFIVLELGGLQDKPDLLVAVMFSLIIYIEDKMYRTPRSLKKCCTIDEGWKLLNFKNEKVGQFIETGYRTVRRHRGAFITISQNIKDFDADDASGAAKAAWGNSAFKVILKQDASEFKQYNQNRPNQFSE
TraCF      AKMVVLELGGLEDRPSLLVAVMFSLIIYIENRMYRTPRNLKKLNVIDEGWRLLDFKNHKVGEFIEKGYRTARRHTGAYITITQNIVDFDSDKASSAARAAWGNSSYKIILKQSAKEFAKYNQLYPDQFLP
consensus  Arm!VLELGGL#DrPdLLVAVMFSLIIYIE#rMYRTPRnLKKcntIDEGWrLL#FKNeKVG#FIEkGYRTaRRHrGA%ITIsQNIkDFDaDdASgAArAAWGNSa%K!ILKQdAkEFaqYNQnrP#QFle

           781     790       800       810       820       830       840       850       860       870   876
           |--------+---------+---------+---------+---------+---------+---------+---------+---------+-----|
TraCED     LERSVIGKFGDAKDQWFSSFMLRINDTCSFHRLFVDPLSRAMFSSKGDDFEFIRQCREQNMDIHDAVYQLAQRNFPDEMRELEALAEAA
TraCF      LQRDMIGKFGAAKDQWFSSFLLQVENHSSWHRLFVDPLSRAMYSSDGPDFEFVQQKRKEGLSIHEAVWQLAWKKSGPEMASLEAWLEEHEKYRSVA
consensus  L#RdmIGKFGaAKDQWFSSF$Lr!##hcSfHRLFVDPLSRAM%SSdGdDFEF!rQcRe#n$dIH#AVwQLAqrnfgdEMaeLEAlaEaa.......
```

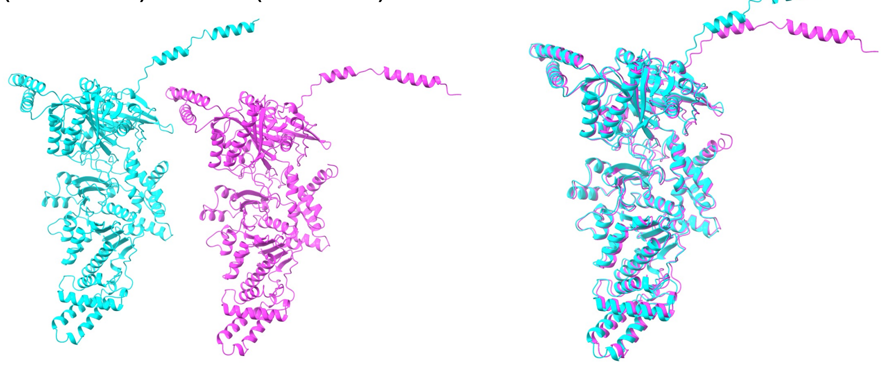

54 % Identity

RMSD: 1.06

TM-score: 0.95

Equivalent residues/
Sequence length:
844/863

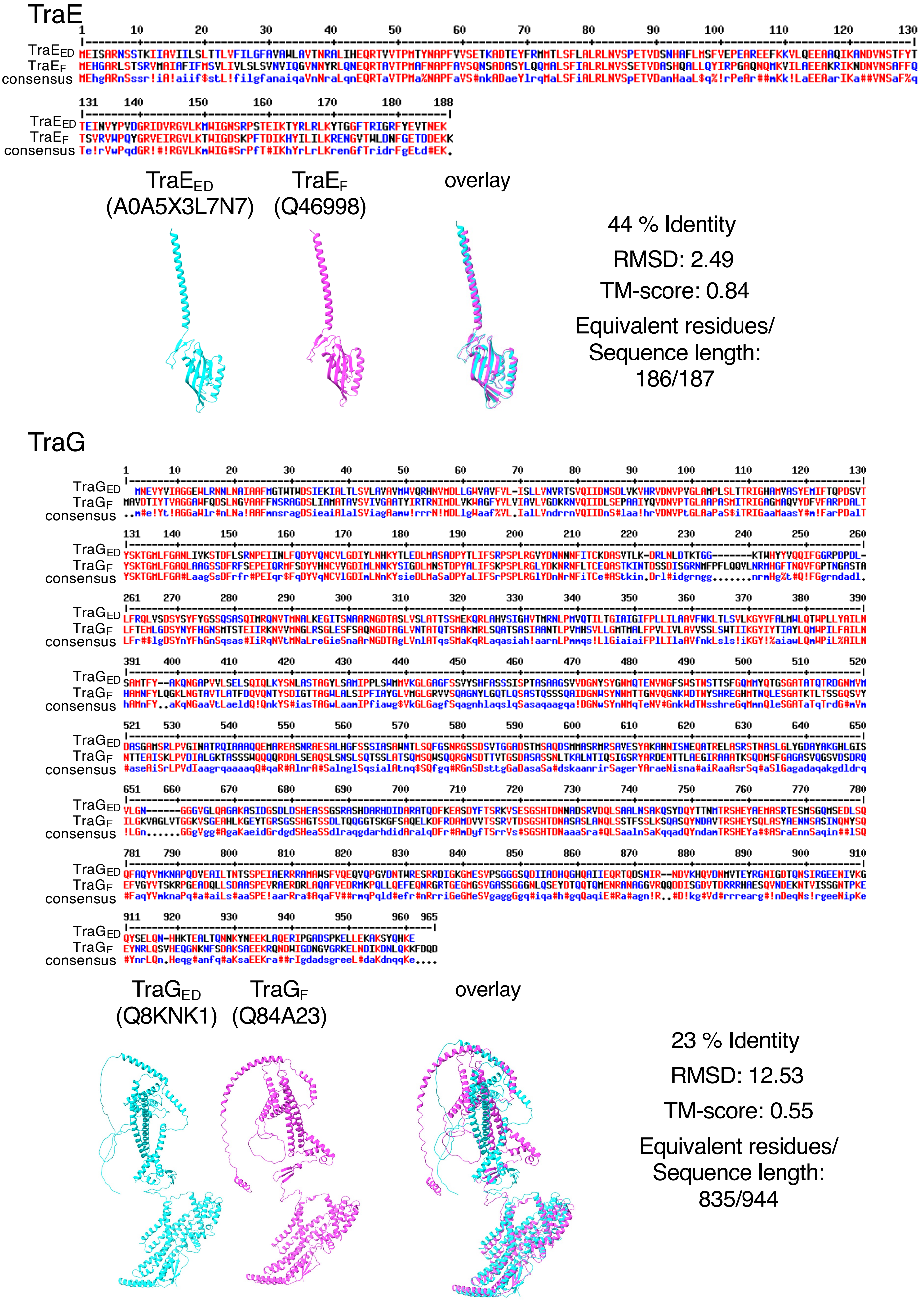

TraE
TraE_ED
TraE_F
consensus
TraE_ED (A0A5X3L7N7)
TraE_F (Q46998)
overlay
44 % Identity
RMSD: 2.49
TM-score: 0.84
Equivalent residues/ Sequence length: 186/187
TraG
TraG_ED
TraG_F
consensus
TraG_ED (Q8KNK1)
TraG_F (Q84A23)
overlay
23 % Identity
RMSD: 12.53
TM-score: 0.55
Equivalent residues/ Sequence length: 835/944

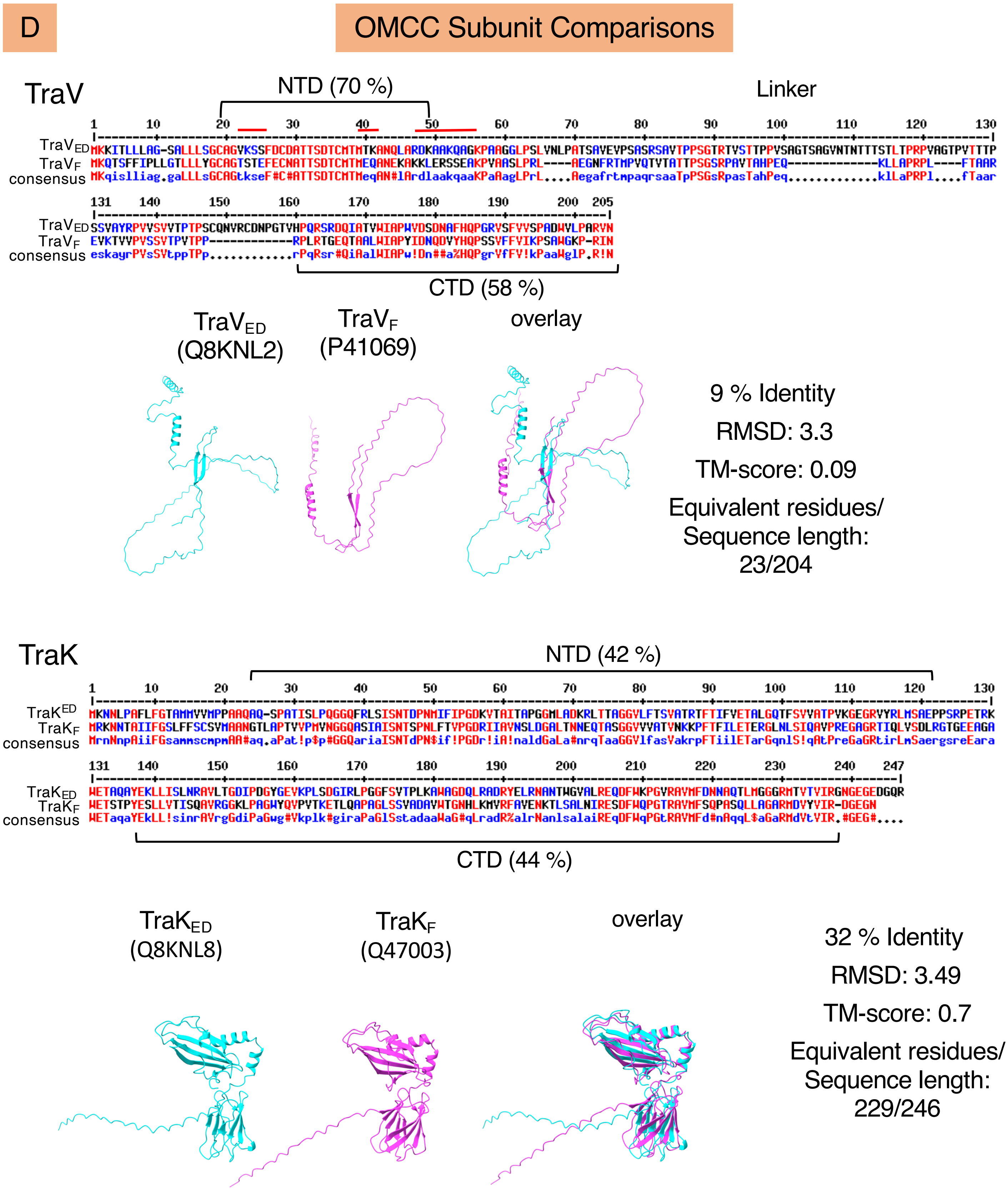
D
OMCC Subunit Comparisons
TraV
NTD (70 %)
Linker
CTD (58 %)
TraVED
TraVF
consensus
TraVED (Q8KNL2)
TraVF (P41069)
overlay
9 % Identity
RMSD: 3.3
TM-score: 0.09
Equivalent residues/ Sequence length: 23/204
TraK
NTD (42 %)
CTD (44 %)
TraKED
TraKF
consensus
TraKED (Q8KNL8)
TraKF (Q47003)
overlay
32 % Identity
RMSD: 3.49
TM-score: 0.7
Equivalent residues/ Sequence length: 229/246

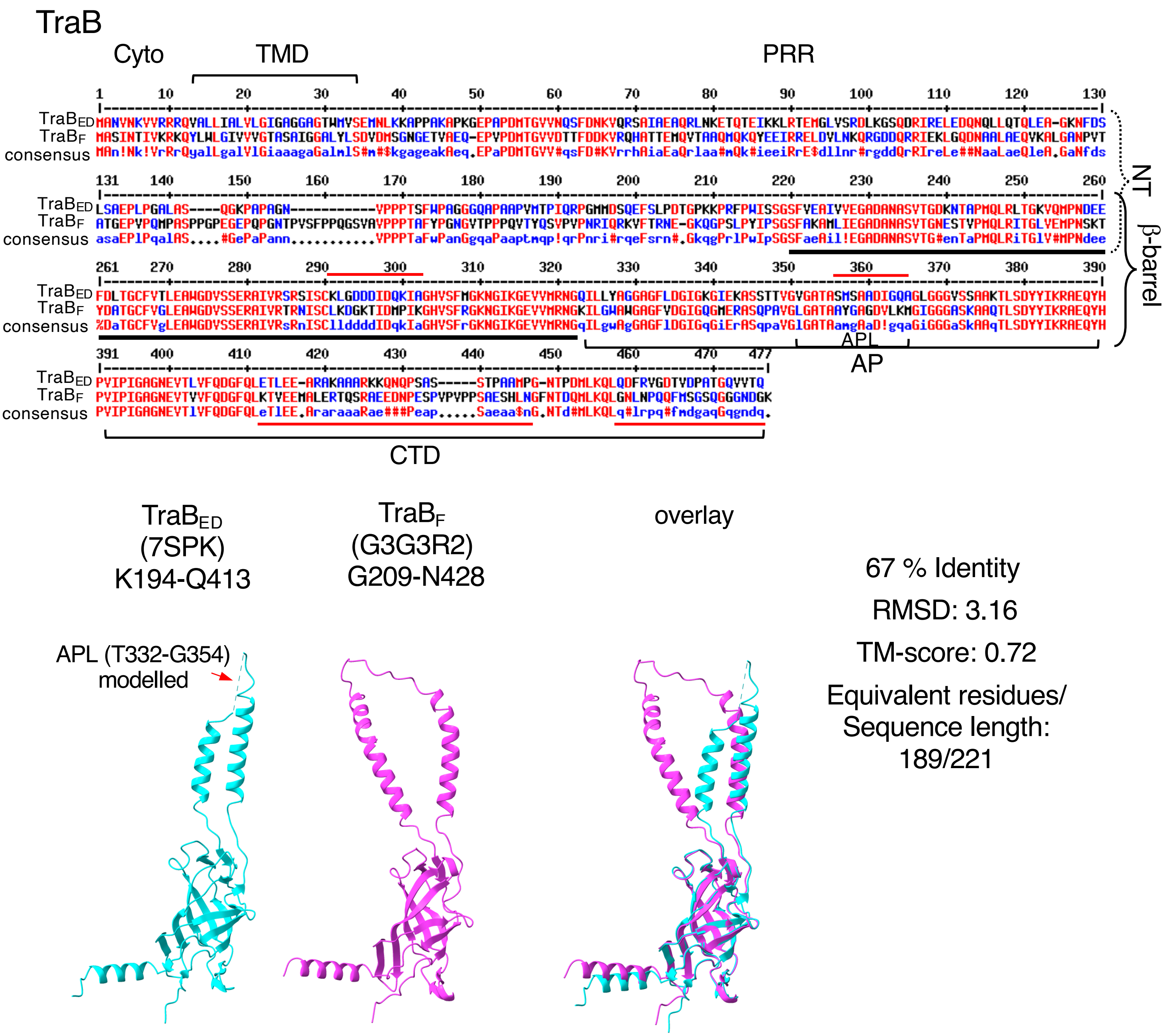

TraB
Cyto
TMD
PRR
NT
β-barrel
APL
AP
CTD
TraB$_{ED}$
TraB$_{F}$
consensus
TraB$_{ED}$ (7SPK) K194-Q413
TraB$_{F}$ (G3G3R2) G209-N428
overlay
APL (T332-G354) modelled
67 % Identity
RMSD: 3.16
TM-score: 0.72
Equivalent residues/ Sequence length: 189/221

E

F-specific Subunit Comparisons

TraF (Class I)

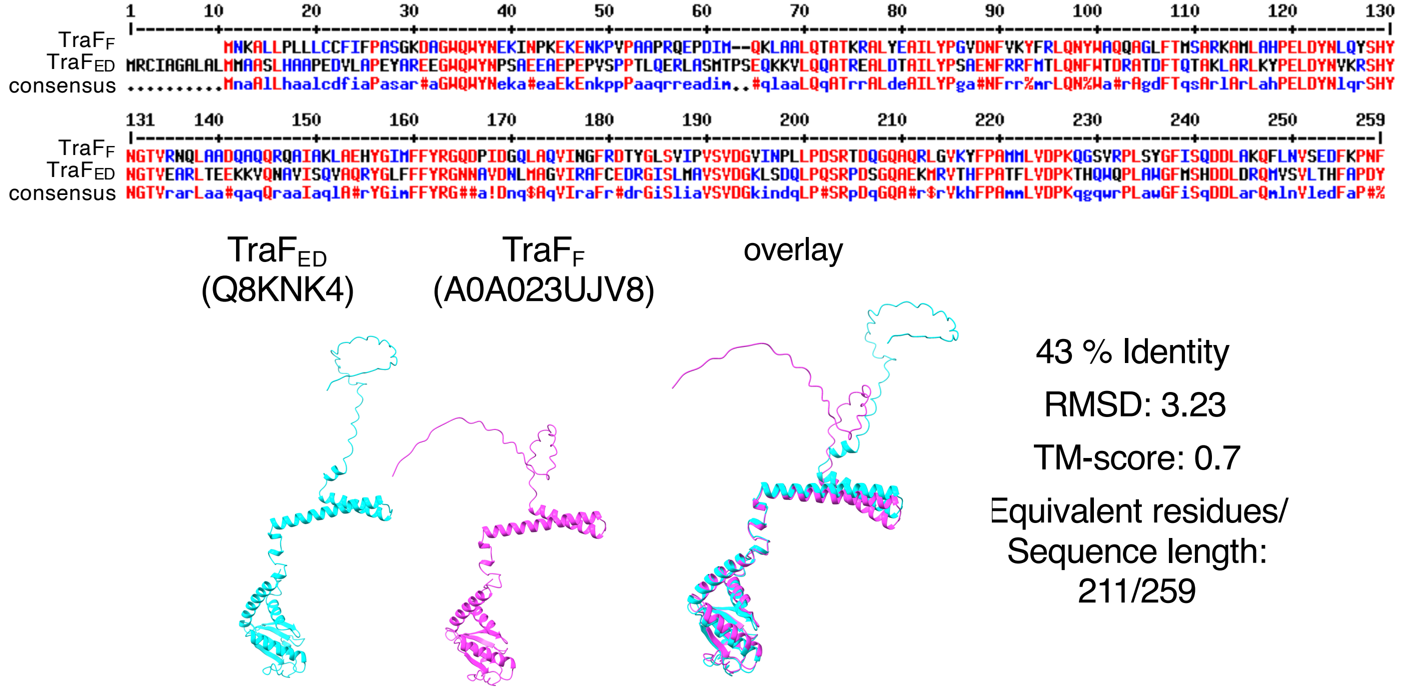

TraH (Class I)

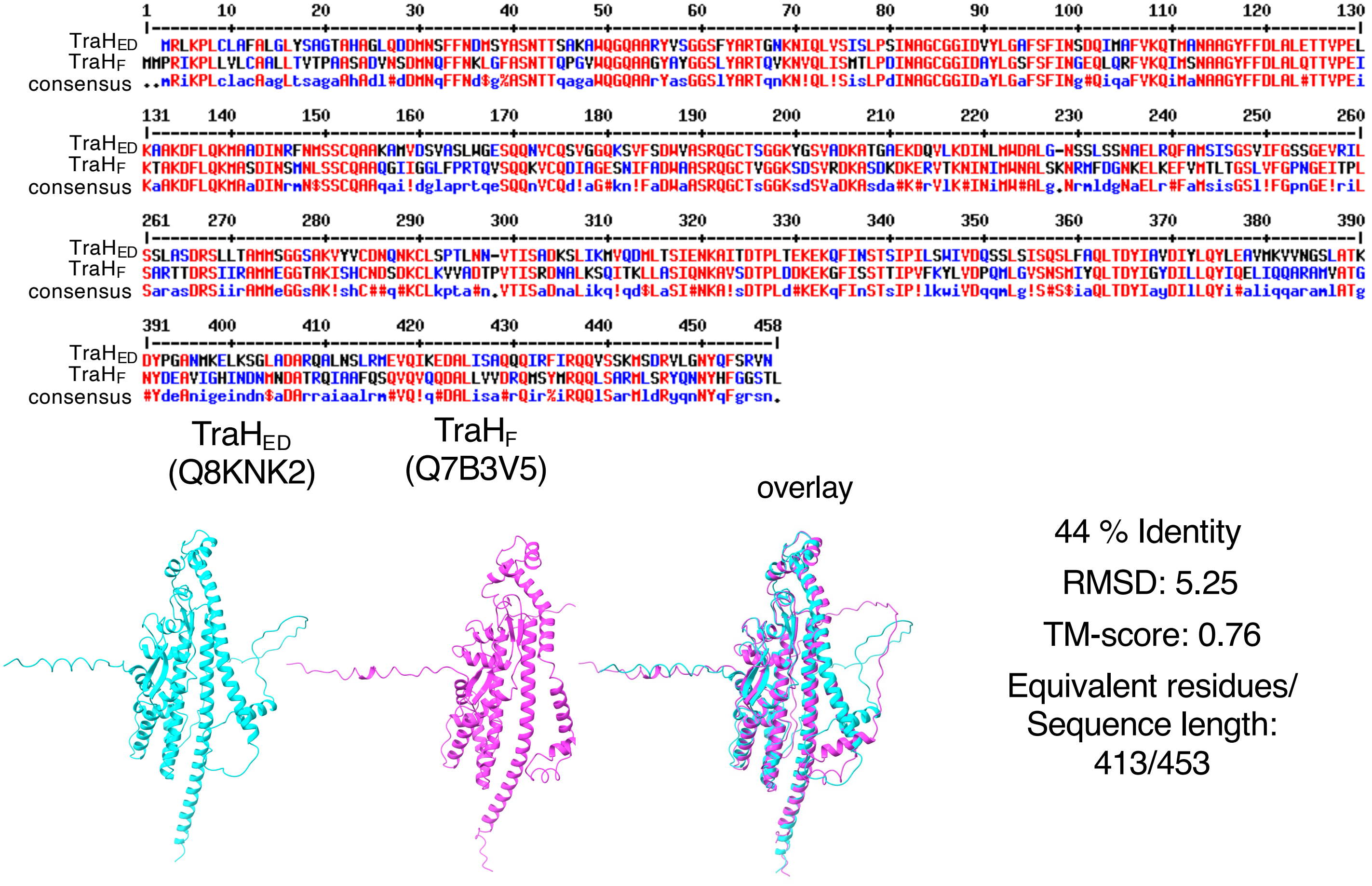

TraW (Class I)

```
              1        10        20        30        40        50        60        70        80        90        100       110       120       130
              |--------+---------+---------+---------+---------+---------+---------+---------+---------+---------+---------+---------+---------|
TraW_ED        MKKGLLTLLLISGVAQAKNLGTWGEMYPIAEQDMLTTIQTRLKAMEASGEMAREQEAFKQRVIENTLRPRPVEGLTLAQENTTHYIDPSLTVSEDLKDHQGRVFAHKGQVINPLDTVPFTDTLYFIDAD
TraW_F        MRCRGLIALLIWGQSVAAADLGTWGDLWPVKEPDMLTVIMQRLTALEQSGEMGRKMDAFKERVIRNSLRPPAVPGIGRTEKYGSRLFDPSVRLAADIRDNEGRVFARQGEVMNPLQYVPFNQTLYFINGD
consensus     .rcrGLiaLLiigqsaaAa#LGTWG#$wP!aEqDMLTtIqqRLkA$EaSGEMaReq#AFK#RVIrNsLRPraVeGigra#engsrliDPSlrlaaDirDn#GRVFArqG#ViNPL#tVPFn#TLYFI#aD

              131      140       150       160       170       180       190       200       21012
              |--------+---------+---------+---------+---------+---------+---------+---------+-|
TraW_ED       NPQQVAWMKAQKPGTLTYRVILVNGDVREATNALNTRIYFDQDGSLCRKFKLKAIPARVTLAEDRRRLRVDTFALDAPEVKP
TraW_F        DPAQVAWMKRQTPPTLESKIILVQGSIPEMQKSLDSRVYFDQNGVLCQRLGIDQVPARVSAVPGDRFLKVEFIPAEEGRK
consensus     #PaQVAWMKaQkPgTLesr!ILV#Gd!rEaqnaL#sR!YFDQ#GsLCrrlgida!PARVsaaedrRrLrV#fiaa#agrk..
```

$TraW_{ED}$ (Q8KNK9) | $TraW_F$ (P18472) | overlay

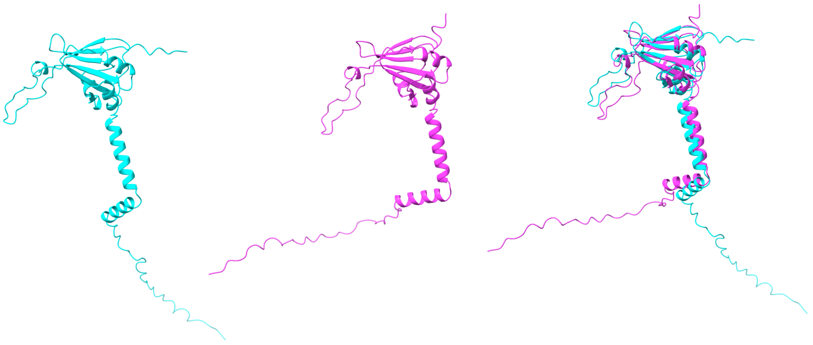

46 % Identity

RMSD: 4.72

TM-score: 0.66

quivalent residues/ Sequence length: 184/211

TraU (Class II)

```
              1        10        20        30        40        50        60        70        80        90        100       110       120       130
              |--------+---------+---------+---------+---------+---------+---------+---------+---------+---------+---------+---------+---------|
TraU_ED       MKRYLAGLGLMLLCLTRMSGTAQAASAECEGNFVNPITDVCWECIFPVTIGNVPVVKGRQPDTPNPSMPIQFCPMG--ILYRVGLAIGYWEPMALTDVTRSPYCMVNLGGFNINIGNVGSGTGGQENRAN
TraU_F        MKRRL---WLLMLFLFAGHVPAASADSACEGRFVNPITDICWSCIFPLSLGSIKVSQGKVPDTANPSMPIQICPAPPPLFRRIGLAIGYWEPMALTDVTRSPGCMVNLGFSLPAFGKTAQGTAKKDEKQV
consensus     MKRrL...gL$$LcLfaghgpAaaAdaaCEGrFVNPITD!CWeCIFPlsiGn!kVsqGrqPDTaNPSMPIQiCPag..ilrR!GLAIGYWEPMALTDVTRSPgCMVNLGgfniaiGntaqGTagq##ran

              131      140       150       160       170       180       190       200       210       220       230       240       250       260
              |--------+---------+---------+---------+---------+---------+---------+---------+---------+---------+---------+---------+---------|
TraU_ED       PGAFYHVHWYKYPLTYWLNIITSMGCLQGGDLDIGYLSELDPMWDSSSLSMIIQPEAILFGNVIAQGACAADAVASLVGKPIDALFWCAGSQGSMYPFNGYVSNEFSPLQTSLLLSERMAFKLHRQGMVM
TraU_F        NGAFYHVHWYKYPLTYWLNIITSLGCLEGGDMDIAYLSEIDPTWTDSSLTTILNPEAVIFANPIAQGACAADAIASAFNMPLDVLFWCAGSQGSMYPFNGWVSNESSPLQSSLLVSERMAFKLHRQGMIM
consensus     nGAFYHVHWYKYPLTYWLNIITS$GCL#GGD$DIaYLSEiDPmWddSSLsmIi#PEA!iFaNpIAQGACAADA!ASafnkPiDaLFWCAGSQGSMYPFNGwVSNEfSPLQsSLLlSERMAFKLHRQGM!M

              261      270       280       290       300       310       320       330333
              |--------+---------+---------+---------+---------+---------+---------+--|
TraU_ED       NSIGENTAVCFEYPSPIIPKSRWRYHLVNKYPDAGQCHPLGRSVTRWETGKNMPNDRRNFGYLFWRKRNCVFL
TraU_F        ETIGKNNAVCNEYPSPILPKERWRYQMVNMYPDSGQCHPFGRSVTRWETGKNPPNTKKNFGYLMWRKRNCVFL
consensus     #sIGeNnAVCnEYPSPIiPKeRWRYq$VNkYPDaGQCHPlGRSVTRWETGKNmPNdrrNFGYLmWRKRNCVFL
```

$TraU_{ED}$ (Q8KNK8) | $TraU_F$ (P18471) | overlay

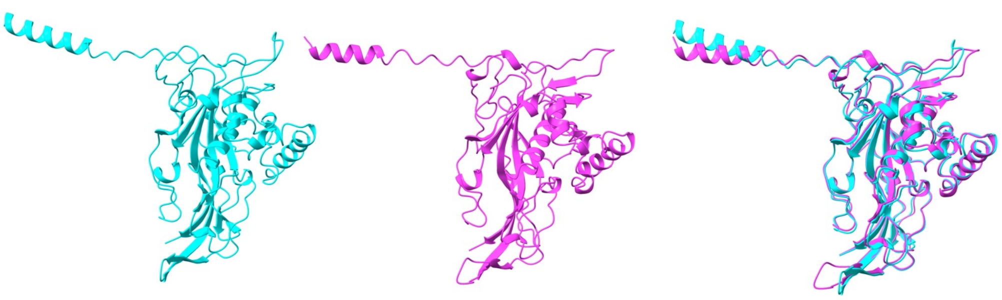

67 % Identity

RMSD: 1.57

TM-score: 0.95

Equivalent residues/ Sequence length: 328/331

TrbC (Class II)

```
          1        10        20        30        40        50        60        70        80        90       100       110       120       130
TrbC_ED   MKRVLISLALLLTC--GAGMASDHT---ENRQFIHDQLQLDRQR-----FRALQTPEFLQQPRPVAPQDQAFLDSQASQFRQAMQPGERPVDAALVFVSFSMPPDELKQRVKDAAELNIPVVIRGMVNGD
TrbC_F    MKLSMKSLAALLMMLNGAVMASENVNTPENRQFLKQQENLSRQLREKPDHQLKAWAEKQVLENPLQRSDNHFLDELVRK-QQASQDG-KPRQGALYFVSFSIPEEGLKRMLGETRHFGIPATLRGMVNND
consensus MKrs$iSLAaLLmc..GAgMAS#nt...ENRQFih#Qe#LdRQr.....hralataEkqqqerPlarqD#aFLDeqarq.rQAmQdG.rPr#aALyFVSFSiPe#eLKrrlg#aaelnIPatiRGMVNnD

          131     140       150       160       170       180       190       200       210    217
TrbC_ED   MRTTANAVAGLVKESNTGGVQIDPTTFRKYNITAVPVLIVACGNQGDTVDRLQGDLTLHQALKRVAEEGDCADTARNLLRGEVQ
TrbC_F    LKTTAEAVLSLVKDGATDGVQIDPTLFSQYGIRTVPALVVFC-SQG--YDIIRGNLRVGQALEKVAATGDCRQVAHDLLAGKGDSGK
consensus $rTTA#AVagLVK#gaTdGVQIDPTlFrqYnIraVPaL!VaC.nQG..yDrirG#LrlgQALerVAaeGDCa#tAr#LLaGeg#...
```

$\mathrm{TrbC_{ED}}$ (Q8KNK6) $\mathrm{TrbC_{F}}$ (P18473) overlay

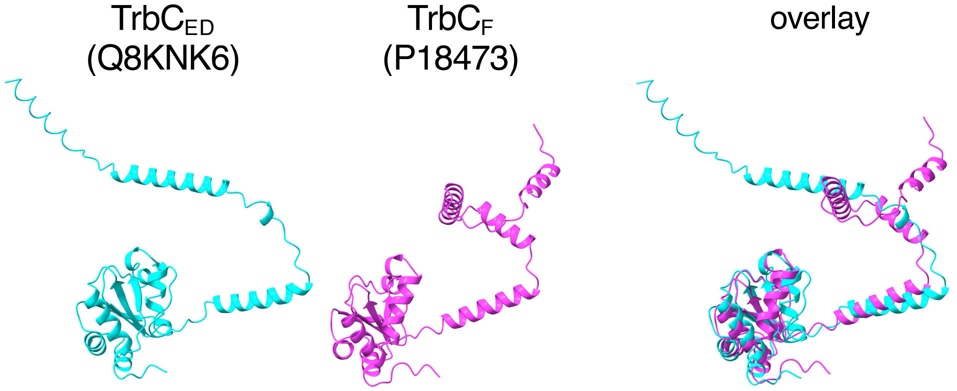

32 % Identity

RMSD: 3.6

TM-score: 0.58

Equivalent residues/ Sequence length: 157/212

TraN (Class II)

```
          1        10        20        30        40        50        60        70        80        90       100       110       120       130
TraN_ED   MKHYLPLLMAMTSFCLVAPLVANDQFNQGMQQGNASKGQGASAIQGFKPAEVIPGYTDSPAESGYYGGVTSSGVD-MTAPGSTALNTSEAGKTITESILNTPPDNKPSLDAPFISEGLAMKDKAETITGG
TraN_F    MKRILPLILALVAG--MAQADSNSDYRAGSDFAHQIKGQGSSSIQGFKPQESIPGYNANPDETKYYGGVTAGGDGGLKNDGTTEWATGETGKTITESFMNKPKDIL-SPDAPFIQTGRDVVNRADSIVGN
consensus MKriLPLi$A$tag..mAqadaNd#%raGm#qanaiKGQGaSaIQGFKPaEsIPGYnanPaEsgYYGGVTagGdd.$kadGsTalaTgEaGKTITESi$NkPkDnl.SlDAPFIqeGramk#rA#sItGn

          131     140       150       160       170       180       190       200       210       220       230       240       250       260
TraN_ED   GFDGCVDQPASFTEITTHQCLRDTKIEQYCTRTATITGDWKNTEVYRYVTISPGQFRYSQNGKQLVFSVTSPVTGVVMNAQLKVYASFYFLNSRYTFMNSVFNVGTVPGATDTFTLSGAPGLNITEGQ-V
TraN_F    TGQQCSAQEISRSEYTNYTCERDLQVEQYCTRTARMELQGSTTWETRTLEYEMSQLPAREVNGQYVVSITSPVTGEIVDA--------HYSWSRTYLQKSVPMTITVLGTPLSWNAKYSADASFTPVQKT
consensus gg#qCsaQeaSrsEiTnhqCeRDlq!EQYCTRTArieg#gknTeetRtleiemgQlrar#nngQlVfS!TSPVTGe!m#A........h%lnSRttlqnSVfntgTVlGapdsfnakgaadaniTegQ.t

          261     270       280       290       300       310       320       330       340       350       360       370       380       390
TraN_ED   LTGSGCTANGNCISGGNGDWQVYQSLASGKSTFTLTMYVRVSEKEWVPRVEWSESCPFSKTEGAMTGSQCVEPGETRTVVVGGKTYSIHQDCWKWQDTYLTQTETEGTCGEFIKNPACTVTRSECAD-TV
TraN_F    LTAGVAFTSSHPVRVGNTKFKRHTAMK-------LRLVVRVKKASYTPYVVWSESCPFSKELGKLTKTECTEAGGNRTLVKDGQSYSMYQSCWAYRDTYVTQSADKGTCQTYTDNPACTLVSHQCAFYSE
consensus LTaggafangnc!rgGNgdfqrhqa$a.......Lr$yVRVkeaewtPrVeWSESCPFSKeeGa$Tgs#CtEaGenRTlVkdGqsYSihQdCWawrDTYlTQsadeGTCqe%idNPACTltrh#CAd.se

          391     400       410       420       430       440       450       460       470       480       490       500       510       520
TraN_ED   DGFCVSQQVTYSCERKKEGNGQICGGEFFCKDGSCAQAQTGTSNMFGQAVSALAAVAAAGEDVAALNGVDVRAFTGEAQHCKKMAVGFNNCCKDSGWGQDVGLSSCSSEEKALGKAKDKKLTVYVGEYCS
TraN_F    EGACLHEYATYSCESKTSGKVMVCGGDVFCLDGECDKAQSGKSNDFAEAVSQLAALAAAGKDVAALNGVDVRAFTGQAKFCKKAAAGYSNCCKDSGWGQDIGLAKCSSDEKALAKAKSNKLTVSVGEFCS
consensus #GaClh#qaTYSCErKkeGngq!CGG#fFClDGeCaqAQsGkSNdFa#AVSaLAAlAAAGeDVAALNGVDVRAFTG#AqhCKKaAaG%nNCCKDSGWGQD!GLakCSS#EKALaKAKdnKLTVsVGE%CS

          521     530       540       550       560       570       580       590       600       610       620
TraN_ED   KKVLGVCLEKKRGYCVFDSKLARIVQEQGRRDQLGVGFGKGKSPDCRGITVDELQRLDFGVMNFSDFYDDLNAGSEIPEDQALLKKAQDIIAEKMQENAP
TraN_F    KKVLGVCLEKKRSYCQFDSKLAQIVQQQGRNGQLRISFGSAKHPDCRGITVDELQKIQFNRLDFTNFYEDLMNNQKIPDSGVLTQKVKEQIADQLKQAGQ
consensus KKVLGVCLEKKRgYCqFDSKLArIVQ#QGRrdQLr!gFGkaKhPDCRGITVDELQri#Fnr$#Fs#FY#DLnanqeIP#dqaLlqKaq#qIA#q$q#aaq
```

ED

$\mathrm{TraN_{ED}}$ (Q8KNK5) $\mathrm{TraN_{F}}$ (P24082) overlay

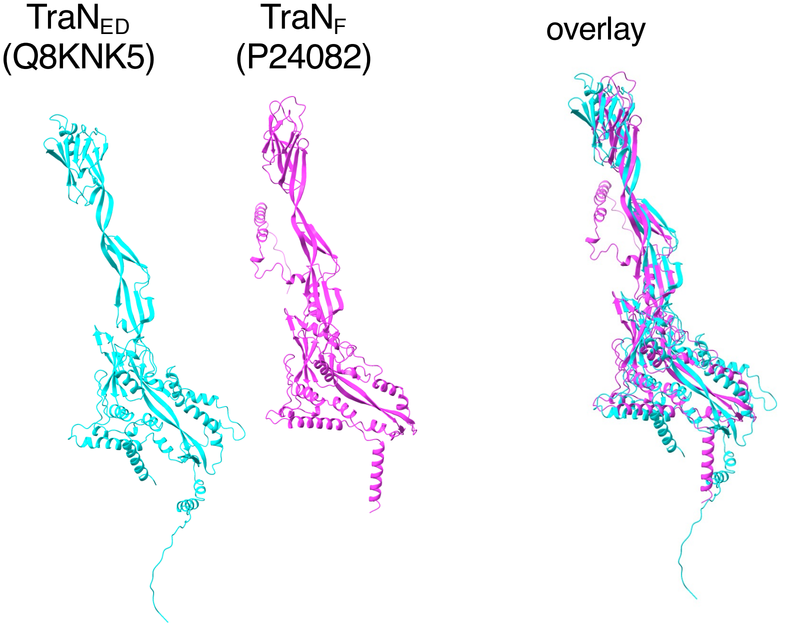

32 % Identity

RMSD: 3.29

TM-score: 0.6

Equivalent residues/ Sequence length: 507/617

TrbB (Class III)

```
            1       10        20        30        40        50        60        70        80        90        100       110       120       130
            |--------+---------+---------+---------+---------+---------+---------+---------+---------+---------+---------+---------+---------|
TrbB_ED     MLITALRTAILMAAMLAGCLQAGTLDDIRALEAGKTGRSAPAMDALSLPENRATPTTDRAAQDTSPVWYTLSDGRKVNLQDWKVVLFMQSTCQYCRQFAPVLKAFSQQTGLDVFPISLDGKGDAEFPDVL
TrbB_F        MSLTKSLLFTLLLSAAAVQASTRDEIERLWNPQGMATQPAQPAAGTSARTAKP---------APRWFRLSNGRQVNLADWKVVLFMQGHCPYCHQFDPVLKQLAQQYGFSVFSYTLDGQGDTAFPEAL
consensus   ..isalrsailmaa$lAaalQAgTrD#IraLeagqggasaPAqdAaglparrAkP.........aPrW%rLS#GRqVNLaDWKVVLFMQghCqYCrQFaPVLKalaQQtGldVFpisLDGqGDaaFP#aL

            131     140       150       160       170       180       190       200203
            |--------+---------+---------+---------+---------+---------+---------+--|
TrbB_ED     PATPDVMVEYFQSGLPVATPTTFLTNVNTMETWPLLQGAAETGEFRSRLDEVLRMALDRQSGKSIPLNAQGQQ
TrbB_F      PVPPDVMQTFFPN-IPVATPTTFLVNVNTLEALPLLQGATDAAGFMARVDTVLQMYGGKKGAK
consensus   PapPDVMqe%Fqn.iPVATPTTFLtNVNT$EalPLLQGAa#aaeFraRlDeVLrMagdrqgaK..........
```

$TrbB_{ED}$ (Q8KNK3)

$TrbB_{F}$ (A0A478LKM9)

overlay

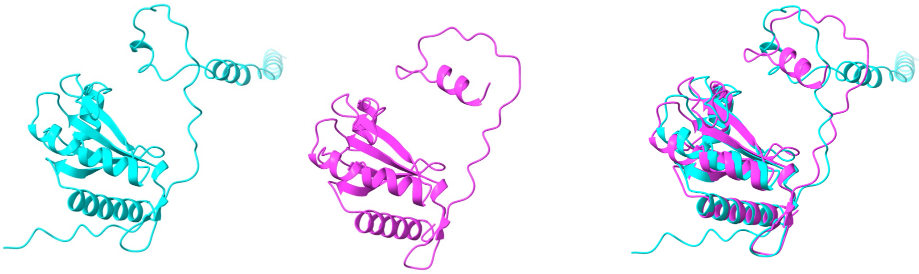

49 % Identity

RMSD: 3.29

TM-score: 0.61

Equivalent residues/ Sequence length: 148/203

TrbI

```
            1       10        20        30        40        50        60        70        80        90        100       110       120       130
            |--------+---------+---------+---------+---------+---------+---------+---------+---------+---------+---------+---------+---------|
TrbI_ED     MTTDQKLTPAQSESTLTSDDVRKGINITAGGHRRERRRRRCLLNLLLVAATIVCINAAVTTLLISWRTPTVVSFDMKKTLDQFTEQASAQSLDEAQTKALTERFMTSLNAELQDWQRRHDALILVTPAVV
TrbI_F                              MSSTQKPADVTAERRSHWWWTVPGCLAMVLLNAAVSYGIVRLNAPVTVAFNMKQTVDAFFDSASQKQLSEAQSKALSARFNTALEASLQAWQQKHHAVILVSPAVV
consensus   ........................iniTaggarrearRRrcllnlllgaaaiVciNAAVstgi!rlraPttVaF#MKqTlDaFf#qASaqqLdEAQsKALsaRFnTaL#AeLQaWQrrHdAlILVsPAVV

            131     140       150 154
            |--------+---------+---|
TrbI_ED     SGAGDITDEIQTGVAHRMAAGGGN
TrbI_F      QGAPDITREIQQDIARRMRAEP
consensus   qGAgDITrEIQqd!ArRMaAeg..
```

$TrbI_{ED}$ (Q8KNL0)

$TrbI_{F}$ (P18006)

overlay

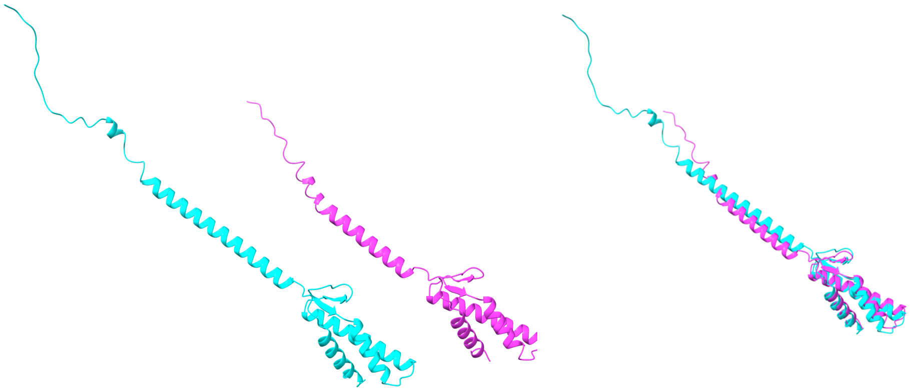

42 % Identity

RMSD: 3.09

TM-score: 0.63

Equivalent residues/ Sequence length: 126/154

F

TraA Pilin Subunit Comparisons

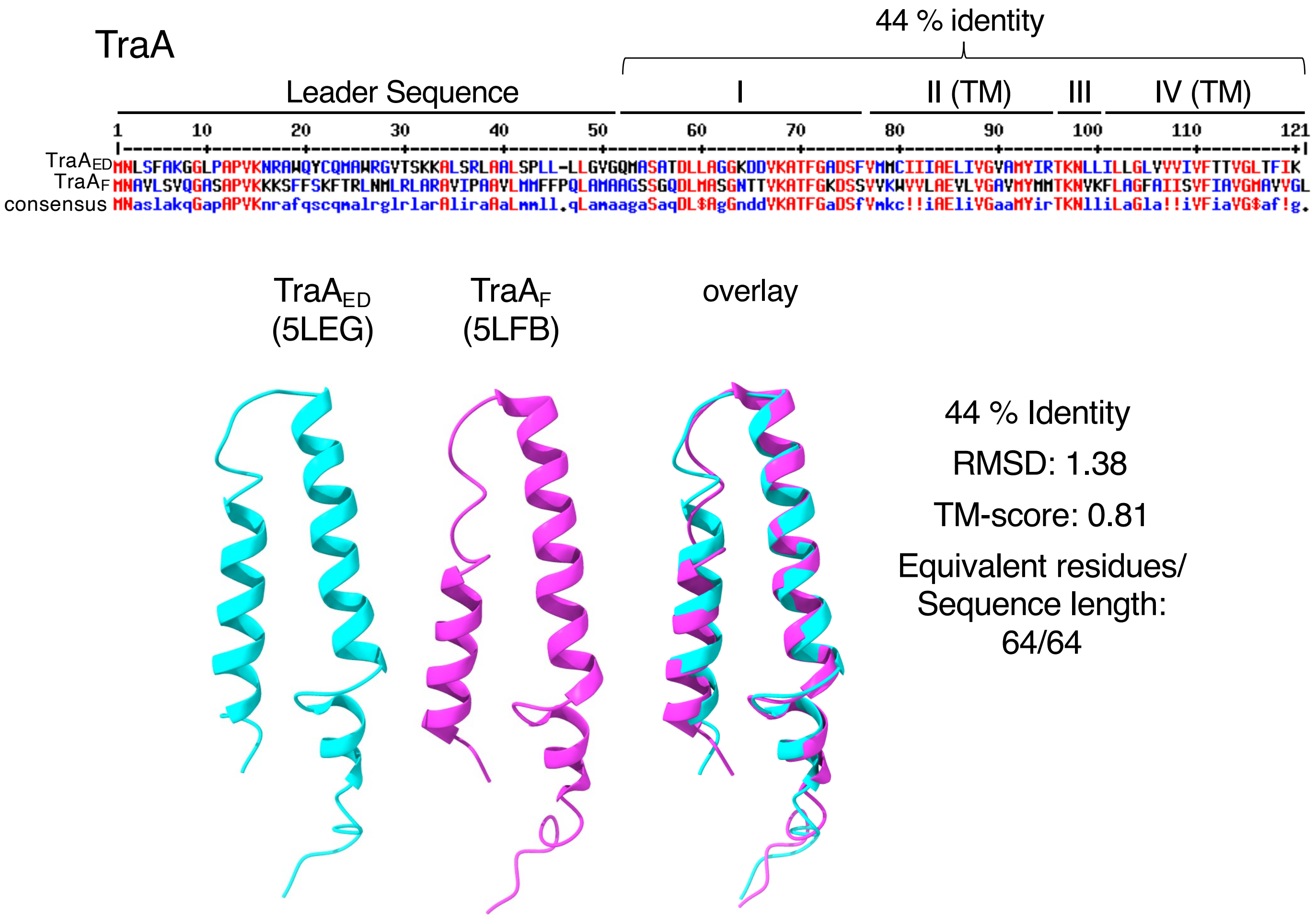

Supplement: S1 Fig — Panel A: Alignment of the F and pED208 tra/trb regions. Blue: VirB/VirD4-like scaffold subunits that are conserved among T4SSs. Red: F-specific proteins that contribute to assembly or function of F-like T4SSs. Yellow: F-specific proteins involved in surface exclusion. Green: Nonconserved proteins. Lines connect genes encoding protein homologs; numbers correspond to the percent amino acid identities across the lengths of the homologs. Accession numbers: pED208 (NCBI Bioproject PRJNA871772); F (NZ_MF370216). By comparison, most F/pED208 VirB/VirD4 subunits exhibit ~18–20% sequence identities with their counterparts in the ‘minimized’ pKM101-encoded system; outliers include the VirB6-like TraGF/TraDKM (8% identical) and VirB7-like TraVF/TraNKM (~6% identical) homologs. Panels B—F (following pages): Similarities among the Tra/Trb homologs at the primary sequence and structural levels, grouped as indicated (orange highlights). Upper: Sequence alignments of the Tra/Trb proteins indicated, generated with Multalin [85]. Known domains and percent identities are shown for regions indicated. Red lines denote regions of sequence divergence, one or more of which might confer the distinct phenotypes accompanying subunit swapping described in the text. Lower: Structures of the Tra/Trb homologs predicted from the AlphaFold Protein Structure Database [82]. The solved TraA and TraBED structures were from RCSB PDB. The structure of pKM101-encoded TraJ was predicted by ColabFold [83]. Structures of the homologs are presented separately and superimposed. For TraB, only the β-barrel and AP domains comprising the OMCC and AP channel are shown. Percent identities of the structurally aligned sequences, Root Mean Square Deviation (RMSD), TM-score, and number of equivalent residues relative to the entire sequence lengths were derived from the RCSB PDB Pairwise Structure Alignment website (https://www.rcsb.org/alignment). (PDF) [file pgen.1011088.s001.pdf]
