# Supplementary material for: Chimeric systems composed of swapped Tra subunits between distantly-related F plasmids reveal striking plasticity among type IV secretion machines
Source: PLoS Genet. 2024 Mar 4;20(3):e1011088. doi: 10.1371/journal.pgen.1011088 (PMC10939261; doi:10.1371/journal.pgen.1011088)
Supplement: S2 Fig — A) Transfer of poriTED and poriTF plasmids by donor cells carrying pED208 (blue bars) or F (green bars). B) Functionality of Strep-TraD in donor cells with pED208 (blue bars) or F (green bars) variants. Donor cells carried pED208 or F or the isogenic ΔtraD mutant plasmids without or with plasmids expressing the corresponding traD or strep-traD genes. Panels A & B) Transfer frequencies are presented as transconjugants per donor (Tcs/D). All matings were repeated at least three times in triplicate; a representative experiment is shown with replicate data points and the average transfer frequencies as horizontal bars along with standard deviations as error bars. Panels C & D) Host cells carrying pED208ΔtraD were assayed for production of the strep-tagged TraDED, TraDF or TraJKM variants shown. Total cellular proteins normalized on a per cell equivalent basis were subjected to SDS-PAGE and immunostaining of western blots with α-strep antibodies for detection of the T4CP variants or α-RNP antibodies against E. coli RNA polymerase β-subunit as a loading control. (PDF) [file pgen.1011088.s002.pdf]

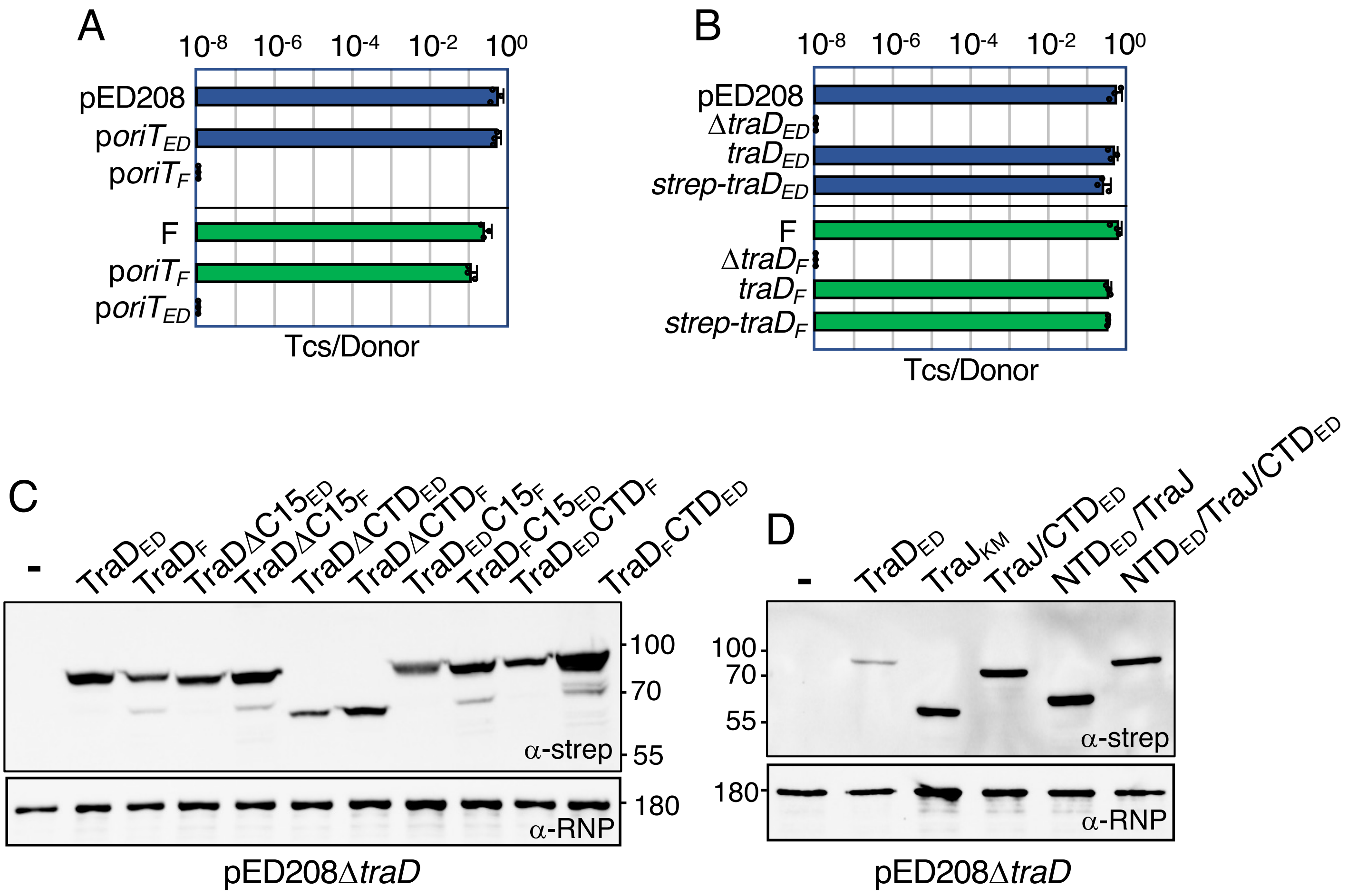

S2 Fig. Functionality of p*oriT* plasmids and *traD* variants. **A)** Transfer of p*oriT*$_{ED}$ and p*oriT*$_{F}$ plasmids by donor cells carrying pED208 (blue bars) or F (green bars). **B)** Functionality of Strep-TraD in donor cells with pED208 (blue bars) or F (green bars) variants. Donor cells carried pED208 or F or the isogenic Δ*traD* mutant plasmids without or with plasmids expressing the corresponding *traD* or *strep-traD* genes. **Panels A & B)** Transfer frequencies are presented as transconjugants per donor (Tcs/D). All matings were repeated at least three times in triplicate; a representative experiment is shown with replicate data points and the average transfer frequencies as horizontal bars along with standard deviations as error bars. **Panels C & D)** Host cells carrying pED208Δ*traD* were assayed for production of the strep-tagged $TraD_{ED}$, $TraD_{F}$ or $TraJ_{KM}$ variants shown. Total cellular proteins normalized on a per cell equivalent basis were subjected to SDS-PAGE and immunostaining of western blots with α-strep antibodies for detection of the T4CP variants or α-RNP antibodies against *E. coli* RNA polymerase β-subunit as a loading control.
