# Supplementary material for: Chimeric systems composed of swapped Tra subunits between distantly-related F plasmids reveal striking plasticity among type IV secretion machines
Source: PLoS Genet. 2024 Mar 4;20(3):e1011088. doi: 10.1371/journal.pgen.1011088 (PMC10939261; doi:10.1371/journal.pgen.1011088)
Supplement: S3 Fig — M13KO7 phage sensitivity is shown for host cells carrying F or pED208 or gene deletion variants without or with a plasmid expressing the corresponding genes from F or pED208. Phage sensitivity is reported as the number of kanamycin-resistant (Kanr) transductants per total colony-forming units (CFUs). Panels: A. traD T4CPs; B. IMC subunits; C. OMCC subunits; D. F-specific components; E. traA pilins. All infection assays were repeated at least three times in triplicate; a representative experiment is shown with replicate data points and the average transfer frequencies as vertical bars along with standard deviations as error bars. Data in the manuscript figures are presented as ‘+’ (sensitive, defined as >10−4 Kanr colonies/total CFUs), ‘-’ (resistant, defined as <10−6 Kanr/total CFUs), or ‘+p’ (partially sensitive, 10−4–6 Kanr/total CFUs). Source data appear in S5 Table. (PDF) [file pgen.1011088.s003.pdf]

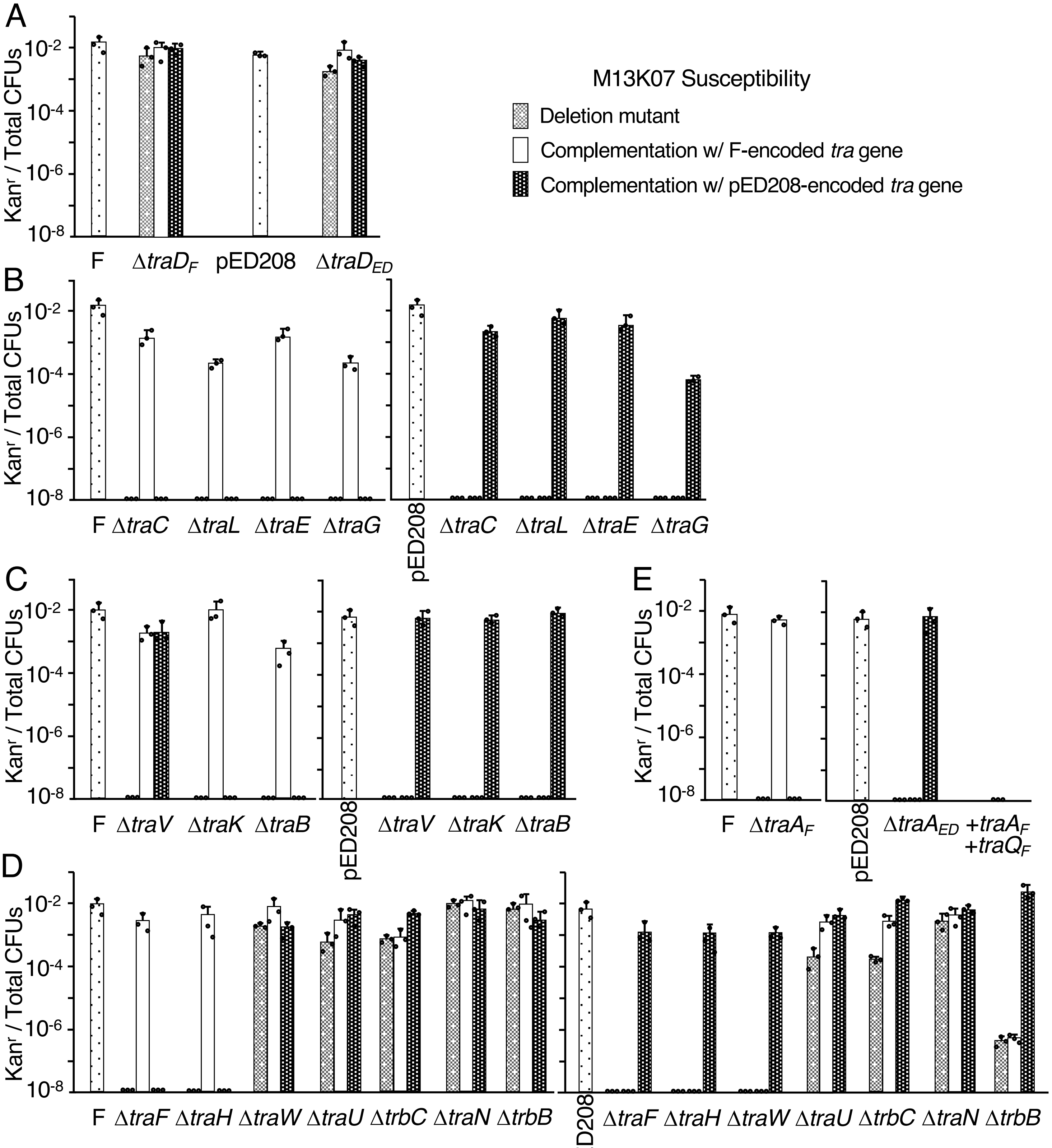

S3 Fig Quantitation of strain sensitivities to M13KO7. M13KO7 phage sensitivity is shown for host cells carrying F or pED208 or gene deletion variants without or with a plasmid expressing the corresponding genes from F or pED208. Phage sensitivity is reported as the number of kanamycin-resistant (Kan^r) transductants per total colony-forming units (CFUs). **Panels A)** *traD* T4CPs; **B)** IMC subunits; **C)** OMCC subunits; **D)** F-specific components; **E)** *traA* pilins. All infection assays were repeated at least three times in triplicate; a representative experiment is shown with replicate data points and the average transfer frequencies as vertical bars along with standard deviations as error bars. Data in the manuscript figures are presented as '+' (sensitive, defined as >10^{-4} Kan^r colonies/total CFUs), '-' (resistant, defined as <10^{-6} Kan^r/total CFUs), or '+^{p}' (partially sensitive, $10^{-4\text{-}6}$ Kan^r/total CFUs). Source data appear in S5 Table.
