# Supplementary material for: Chimeric systems composed of swapped Tra subunits between distantly-related F plasmids reveal striking plasticity among type IV secretion machines
Source: PLoS Genet. 2024 Mar 4;20(3):e1011088. doi: 10.1371/journal.pgen.1011088 (PMC10939261; doi:10.1371/journal.pgen.1011088)
Supplement: S4 Fig — Transfer of donors harboring Tra+,Pil- “uncoupling” mutations in liquid matings (A) and production of TraB variants and TraAED pilin in different mutant backgrounds (B-D). A) pED208 or F transfer frequencies by donors bearing “uncoupling” mutations in 1 h liquid matings; matings were repeated at least three times in triplicate and a representative experiment is shown with the average transfer frequencies as horizontal bars along with standard deviations as error bars. Colored bars denote source of plasmid or expressed gene of interest (blue, pED208; green, F; chimera, blue/green checkered); for comparison, gray bars represent transfer frequencies for the same strains in solid-surface matings as reported in the manuscript. B) Schematics of TraB chimeras and deletion mutants. Chimeras consist of domains from TraBED (blue) and TraBF (green). Host cells carrying pED208ΔtraB and complementing plasmids were assayed for production of the strep-tagged TraB variants shown. Total cellular proteins normalized on a per cell equivalent basis were subjected to SDS-PAGE and immunostaining of western blots with α-strep antibodies for detection of the TraB variants or α-RNP antibodies against E. coli RNP β-subunit as a loading control. C) Production of TraAED pilin in strains carrying pED208 or mutant plasmids deleted of the tra/trb genes shown; Lane 2: E. coli MC4100 expressing traAED from pKKF004 in the absence of pED208. D) Schematic of the pED208 tra/trb region with the ΔOMCC and ΔF-specific deletion mutations highlighted; production of TraAED pilin in strains with the pED208ΔOMCC and pED208ΔF-specific mutant plasmids. Panels C & D) TraAED pilin was detected by immunostaining with α-TraAED polyclonal antibodies (upper and lower bands are crossreactive species) and RNP β-subunit with α-RNP antibodies. (PDF) [file pgen.1011088.s004.pdf]

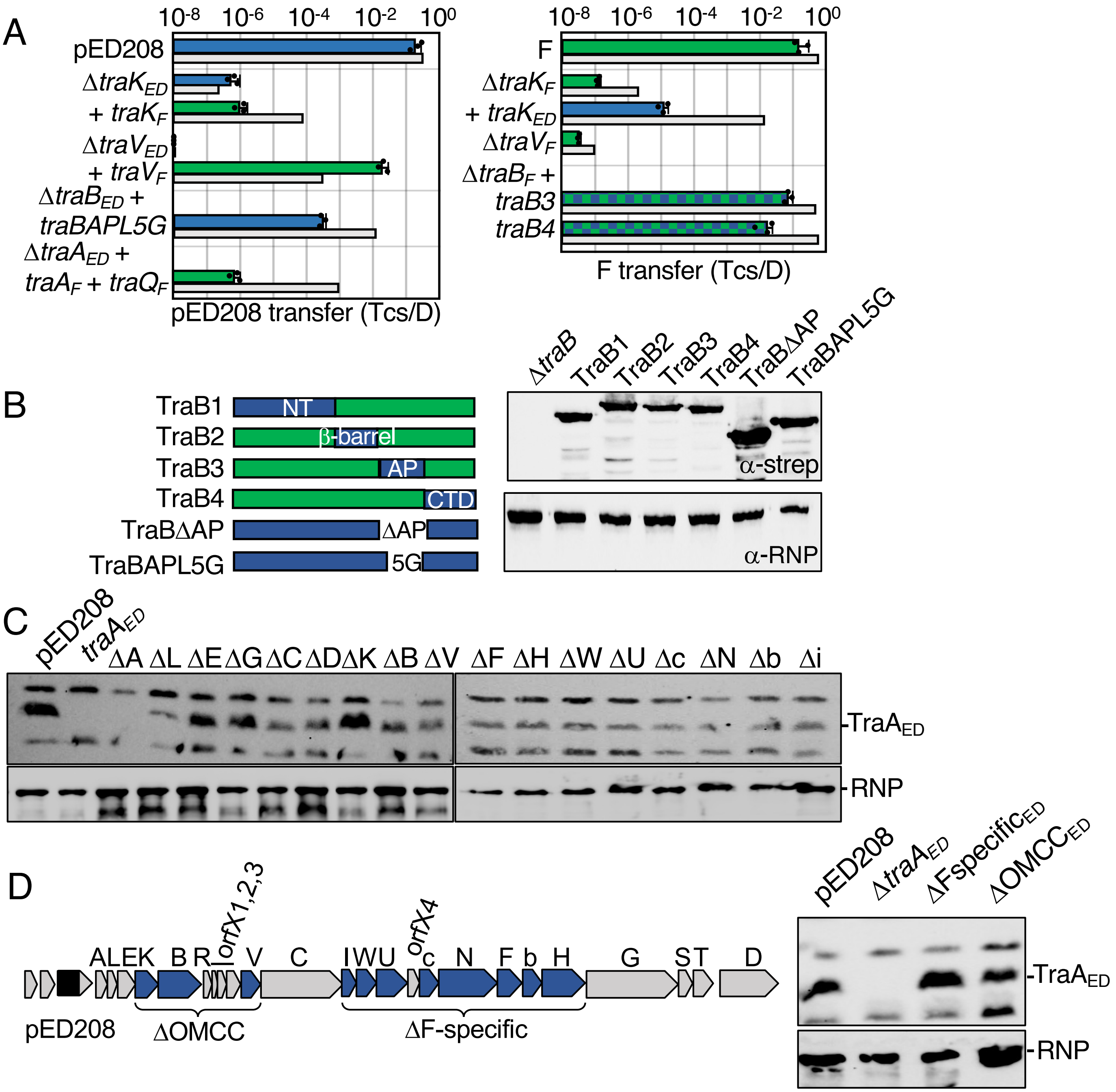

S4 Fig. Transfer of donors harboring Tra+,Pil- "uncoupling" mutations in liquid matings (A) and production of TraB variants and TraA$_{ED}$ pilin in different mutant backgrounds (B-D). A) pED208 or F transfer frequencies by donors bearing "uncoupling" mutations in 1 h liquid matings; matings were repeated at least three times in triplicate and a representative experiment is shown with the average transfer frequencies as horizontal bars along with standard deviations as error bars. Colored bars denote source of plasmid or expressed gene of interest (blue, pED208; green, F; chimera, blue/green checkered); for comparison, gray bars represent transfer frequencies for the same strains in solid-surface matings as reported in the manuscript. **B)** Schematics of TraB chimeras and deletion mutants. Chimeras consist of domains from TraB$_{ED}$ (blue) and TraB$_{F}$ (green). Host cells carrying pED208Δ*traB* and complementing plasmids were assayed for production of the strep-tagged TraB variants shown. Total cellular proteins normalized on a per cell equivalent basis were subjected to SDS-PAGE and immunostaining of western blots with α-strep antibodies for detection of the TraB variants or α-RNP antibodies against *E. coli* RNP β-subunit as a loading control. **C)** Production of TraA$_{ED}$ pilin in strains carrying pED208 or mutant plasmids deleted of the *tra/trb* genes shown; Lane 2: *E. coli* MC4100 expressing *traA$_{ED}$* from pKKF004 in the absence of pED208. **D)** Schematic of the pED208 *tra/trb* region with the ΔOMCC and ΔF-specific deletion mutations highlighted; production of TraA$_{ED}$ pilin in strains with the pED208ΔOMCC and pED208ΔF-specific mutant plasmids. **Panels C & E)** TraA$_{ED}$ pilin was detected by immunostaining with α-TraA$_{ED}$ polyclonal antibodies (upper and lower bands are crossreactive species) and RNP β-subunit with α-RNP antibodies.
