# Supplementary material for: Chimeric systems composed of swapped Tra subunits between distantly-related F plasmids reveal striking plasticity among type IV secretion machines
Source: PLoS Genet. 2024 Mar 4;20(3):e1011088. doi: 10.1371/journal.pgen.1011088 (PMC10939261; doi:10.1371/journal.pgen.1011088)
Supplement: S1 Table — (PDF) [file pgen.1011088.s005.pdf]

Table S1. Strains and plasmids used in this study.

Strains or plasmids	Relevant characteristics	Source
DH5α	F$^-$ *endA1 glnV44 thi-1 recA1 relA1 gyrA96 deoR nupG purB20* φ80*dlacZ*ΔM15 Δ(*lacZYA-argF*)U169, hsdR17(r_K^- m_K^+), λ$^-$	Lab stock
MC4100	F$^-$ [*araD139*]$_{B/r}$ Δ(*argF-lac*)*169* λ$^-$ *e14*$^-$ *flhD5301 Δ(fruK-yeiR)725(fruA25) relA1 rpsL150(strR) rbsR22 Δ(fimB-fimE)632(::IS1) deoC*	Lab stock
AA116	MC4100::*rif*^{R} *Cm*^{R}	[1]
MG1655	K-12 F$^-$ λ$^-$ *ilvG*$^-$ *rfb-50 rph-1*	Lab stock
HME45-RifR	W3110 *gal490 pgl*Δ*8* λ*cI857* Δ*(cro-bioA) rif*^{R}	[2]
<u>Plasmid vectors</u>		
pBAD24	*crb*^{R}, P_{BAD} cloning vector	[3]
pKG116	pACYC184 derivative with *nahG* promoter and RBS	[4]
pBAD101	*spc*^{R}, pSC101 with P_{BAD} promoter	[5]
pCP20	Yeast Flp recombinase on a temperature-sensitive replicon	[6]
pKD13	*crb*^{R}, R6K plasmid with FRT-*kan*$^{R-}$FRT	[6]
pKD46	*crb*^{R}, λ-Red recombinase expression plasmid	[6]
<u>pED208 variants</u>		
pED208-Spc	*spc*^{R}, IncFV derivative of F_0*lac*	[2]
pED208-Tc	*tet*^{R}, IncFV derivative of F_0*lac*	[7]
pED208Δ*traA*	pED208-Spc deleted of *traA*	[2]
pED208Δ*traB*	pED208-Spc deleted of *traB*	[2]
pED208Δ*traK*	pED208-Spc deleted of *traK*	[7]
pED208Δ*traV*	pED208-Spc deleted of *traV*	[7]
pED208ΔOMCC	pED208-Spc deleted of *traB-traV*	This study
pED208Δ*traL*	pED208-Spc deleted of *traL*	This study
pED208Δ*traE*	pED208-Spc deleted of *traE*	This study
pED208Δ*traC*	pED208-Spc deleted of *traC*	[2]
pED208Δ*traD*	pED208-Spc deleted of *traD*	[2]
pED208Δ*traG*	pED208-Spc deleted of *traG*	This study
pED208Δ*trbI*	pED208-Spc deleted of *trbI*	[8]
pED208Δ*traW*	pED208-Spc deleted of *traW*	[8]
pED208Δ*traU*	pED208-Spc deleted of *traU*	[8]
pED208Δ*trbC*	pED208-Spc deleted of *trbC*	[8]
pED208Δ*traN*	pED208-Spc deleted of *traN*	[8]
pED208Δ*traF*	pED208-Spc deleted of *traF*	[8]
pED208Δ*trbB*	pED208-Spc deleted of *trbB*	[8]
pED208Δ*traH*	pED208-Spc deleted of *traH*	[8]
pED208ΔFspecific	pED208-Spc deleted of *trbI-traH*	This study
pYGL249	pBAD101 carrying pED208 *oriT* sequence	This study
<u>pOX38 (F) variants</u>		
pOX38-Tc	*tet*^{R}, F deleted of HindIII fragment	[9]
pOX38Δ*traA*	pOX38-Tet deleted of *traA*	[8]
pOX38Δ*traB*	pOX38-Tet deleted of *traB*	This study
pOX38Δ*traV*	pOX38-Tet deleted of *traV*	This study
pOX38Δ*traK*	pOX38-Tet deleted of *traK*	This study
pOX38Δ*traL*	pOX38-Tet deleted of *traL*	This study
pOX38Δ*traE*	pOX38-Tet deleted of *traE*	This study
pOX38Δ*traC*	pOX38-Tet deleted of *traC*	This study
pOX38Δ*traD*	pOX38-Tet deleted of *traD*	This study
pOX38Δ*traG*	pOX38-Tet deleted of *traG*	This study
pOX38Δ*trbI*	pOX38-Tet deleted of *trbi*	[8]
pOX38Δ*traW*	pOX38-Tet deleted of *traW*	[8]
pOX38Δ*traU*	pOX38-Tet deleted of *traU*	[8]
pOX38Δ*trbC*	pOX38-Tet deleted of *trbC*	[8]
pOX38Δ*traN*	pOX38-Tet deleted of *traN*	[8]

pOX38Δ*traF*	pOX38-Tet deleted of *traF*	[8]
pOX38Δ*trbB*	pOX38-Tet deleted of *trbB*	[8]
pOX38Δ*traH*	pOX38-Tet deleted of *traH*	[8]
pOX38Km-traQ238	pOX38-Tet deleted of *traQ*::Km^R marker	[10]
pOX38ΔFspecific	pOX38-Tet deleted of *trbI-traH*	This study
pYGL248	pBAD101 carrying F *oriT* sequence	This study
tra gene expression plasmids (pED208)		
pKKF004	chl^R, pKG116 expressing $traA_{ED}$	[8]
pKKF005	chl^R, pKG116 expressing $traA.T116C_{ED}$	[8]
pPK019	crb^R, pBAD24 expressing $traB_{ED}$	[2]
pKN9	crb^R, pBAD24 expressing *traB1*	This study
pKN10	crb^R, pBAD24 expressing *traB2*	This study
pKN11	crb^R, pBAD24 expressing *traB3*	This study
pKN12	crb^R, pBAD24 expressing *traB4*	This study
pPK020	crb^R, pBAD24 expressing $traB\Delta AP_{ED}$	This study
pPK021	crb^R, pBAD24 expressing *traBAPL5G*	This study
pYGL683	crb^R, pBAD24 expressing $traB\Delta AP_{strep}$	This study
pYGL684	crb^R, pBAD24 expressing $traBAPL5G_{strep}$	This study
pPK022	crb^R, formerly pBAD24-$traK_{ED}$	[7]
pPK023	crb^R, formerly pBAD24-$traV_{ED}$	[7]
pAM3	crb^R, pBAD24 expressing $traL_{ED}$	This study
pAM4	crb^R, pBAD24 expressing $traE_{ED}$	This study
pPK015	crb^R, pBAD24 expressing $traC_{ED}$	[2]
pPK018	crb^R, pBAD24 expressing $traD_{ED}$	[2]
pAM1	crb^R, pBAD24 expressing $traG_{ED}$	This study
pRBF001	crb^R, pBAD24 expressing $trbI_{ED}$	[8]
pRBF002	crb^R, pBAD24 expressing $traW_{ED}$	[8]
pRBF003	crb^R, pBAD24 expressing $traU_{ED}$	[8]
pKKF001	crb^R, pBAD24 expressing $trbC_{ED}$	[8]
pPKF001	crb^R, pBAD24 expressing $traN_{ED}$	[8]
pRBF004	crb^R, pBAD24 expressing $traF_{ED}$	[8]
pKKF002	crb^R, pBAD24 expressing $trbB_{ED}$	[8]
pRBF005	crb^R, pBAD24 expressing $traH_{ED}$	[8]
pYGL343	chl^R, pKG116 expressing ${}_{strep}traD_{ED}$	This study
pYGL351	chl^R, pKG116 expressing ${}_{strep}traD\Delta C15_{ED}$	This study
pYGL353	chl^R, pKG116 expressing ${}_{strep}traD_{ED}C15_{F}$	This study
pYGL491	chl^R, pKG116 expressing ${}_{strep}traD\Delta C166_{ED}$	This study
tra gene expression plasmids (F)		
pKKF083	chl^R, pKG116 expressing $traA_F$	This study
pRBF013	crb^R, formerly pBAD24-$traB_F$	This study
pRBF014	crb^R, pBAD24 expressing $traK_F$	This study
pRPF015	crb^R, pBAD24 expressing $traV_F$	This study
pKKF079	crb^R, pBAD24 expressing $traL_F$	This study
pKKF078	crb^R, pBAD24 expressing $traE_F$	This study
pPKF024	crb^R, pBAD24 expressing $traC_F$	This study
pPKF025	crb^R, pBAD24 expressing $traD_F$	This study
pPKF026	crb^R, pBAD24 expressing $traG_F$	This study
pRBF006	crb^R, pBAD24 expressing $trbI_F$	[8]
pRBF007	crb^R, pBAD24 expressing $traW_F$	[8]
pRBF008	crb^R, pBAD24 expressing $traU_F$	[8]
pKKF003	crb^R, pBAD24 expressing $trbC_F$	[8]
pRBF009	crb^R pBAD24 expressing $traN_F$	[8]
pRBF010	crb^R, pBAD24 expressing $traF_F$	[8]
pRBF011	crb^R, pBAD24 expressing $trbB_F$	[8]
pRBF012	crb^R, pBAD24 expressing $traH_F$	[8]
pKN3	crb^R, pBAD24 expressing $traQ_F$	This study

pYGL342	*chl*^{R}, pKG116 expressing $_{strep}$*traD*$_F$	This study
pYGL348	*chl*^{R}, pKG116 expressing $_{strep}$*traDΔC15*$_F$	This study
pYGL352	*chl*^{R}, pKG116 expressing $_{strep}$*traD*$_F$*C15*$_{ED}$	This study
pYGL492	*chl*^{R}, pKG116 expressing $_{strep}$*traDΔC148*$_F$	This study
tra gene expression plasmids (pKM101)		
pYGL493	*chl*^{R}, pKG116 expressing *traJ*$_{KMstrep}$	This study
pYGL494	*chl*^{R}, pKG116 expressing *traJ*$_{KMstrep}$*C166*$_{ED}$	This study
pYGL528	*chl*^{R}, pKG116 expressing *N1-134*$_{ED}$*traJ*$_{KMstrep}$	This study
pYGL529	*chl*^{R}, pKG116 expressing *N1-134traJ*$_{KMstrep}$*C166*$_{ED}$	This study
pCGR97	*crb*^{R}, pBAD24 expressing *oriT-traK-traJ-traI*$_{KM}$	This study
pYGL490	*crb*^{R}, pBAD24 expressing *oriT-traK-traI*$_{KM}$	This study

References.

1. Al Mamun AAM, Kishida K, Christie PJ. Protein transfer through an F plasmid-encoded type IV secretion system suppresses the mating-induced SOS response. mBio. 2021;12(4):e0162921. Epub 2021/07/14. doi: 10.1128/mBio.01629-21. PubMed PMID: 34253063.
2. Hu B, Khara P, Christie PJ. Structural bases for F plasmid conjugation and F pilus biogenesis in *Escherichia coli.* Proc Natl Acad Sci U S A. 2019;116(28):14222-7. Epub 2019/06/27. doi: 10.1073/pnas.1904428116. PubMed PMID: 31239340; PubMed Central PMCID: PMCPMC6628675.
3. Guzman LM, Belin D, Carson MJ, Beckwith J. Tight regulation, modulation, and high-level expression by vectors containing the arabinose PBAD promoter. J Bacteriol. 1995;177(14):4121-30.
4. Haeusser DP, Rowlett VW, Margolin W. A mutation in *Escherichia coli ftsZ* bypasses the requirement for the essential division gene *zipA* and confers resistance to FtsZ assembly inhibitors by stabilizing protofilament bundling. Mol Microbiol. 2015;97(5):988-1005. Epub 2015/06/06. doi: 10.1111/mmi.13081. PubMed PMID: 26046682; PubMed Central PMCID: PMCPMC4641749.
5. Li YG, Christie PJ. The TraK accessory factor activates substrate transfer through the pKM101 type IV secretion system independently of its role in relaxosome assembly. Mol Microbiol. 2020;114(2):214-29. Epub 2020/04/03. doi: 10.1111/mmi.14507. PubMed PMID: 32239779; PubMed Central PMCID: PMCPMC7529830.
6. Datsenko KA, Wanner BL. One-step inactivation of chromosomal genes in *Escherichia coli* K-12 using PCR products. Proc Natl Acad Sci U S A. 2000;97(12):6640-5. Epub 2000/06/01. doi: 10.1073/pnas.120163297. PubMed PMID: 10829079; PubMed Central PMCID: PMCPMC18686.
7. Liu X, Khara P, Baker ML, Christie PJ, Hu B. Structure of a type IV secretion system core complex encoded by multi-drug resistance F plasmids. Nat Commun. 2022;13(1):379. Epub 2022/01/21. doi: 10.1038/s41467-022-28058-5. PubMed PMID: 35046412; PubMed Central PMCID: PMCPMC8770708.
8. Kishida K, Bosserman RE, Harb L, Khara P, Song L, Hu B, et al. Contributions of F-specific subunits to the F plasmid-encoded type IV secretion system and F pilus. Mol Microbiol. 2022;117(5):1275-90. Epub 2022/04/19. doi: 10.1111/mmi.14908. PubMed PMID: 35434837; PubMed Central PMCID: PMCPMC9359479.
9. Anthony KG, C. Sherburne, R. Sherburne, and L. S. Frost. The role of the pilus in recipient cell recognition during bacterial conjugation mediated by F-like plasmids. Mol Microbiol. 1994;13:939-53.
10. Kathir P, Ippen-Ihler K. Construction and characterization of derivatives carrying insertion mutations in F plasmid transfer region genes, *trbA*, *artA*, *traQ*, and *trbB*. Plasmid. 1991;26(1):40-54. Epub 1991/07/01. PubMed PMID: 1658835.
