# Supplementary material for: Chimeric systems composed of swapped Tra subunits between distantly-related F plasmids reveal striking plasticity among type IV secretion machines
Source: PLoS Genet. 2024 Mar 4;20(3):e1011088. doi: 10.1371/journal.pgen.1011088 (PMC10939261; doi:10.1371/journal.pgen.1011088)
Supplement: S2 Table — (PDF) [file pgen.1011088.s006.pdf]

Table S2. Oligonucleotides used in this study.

Primer name	Sequence (5' to 3')	Purpose (plasmid constructions & confirmations)
AA5_traL_delF	GGTTGTGGTTATCGTCTTCACTACCGTCGGTCTTACCT TCATCAAATGATATGATTCCGGGGATCCGTCGACC	pED208Δ*traL*
AA6_traL_delR	CGCTAATTTCCATAATATGGCCCTGCGGTAATTTATTT TATCCATTTCCTGAATGTAGGCTGGAGCTGCTTCG	pED208Δ*traL*
AA29_traLEchk_F	GTGCATGGCAGTACTGCCAG	pED208Δ*traL* and pED208Δ*traE*
AA30_traLEchk_R	CCTTCTCCTT TCACCGGAGT G	pED208Δ*traL* and pED208Δ*traE*
AA7_traE_delF	CGATTCCAGTTTCAGGAAATGGATAAAATAAATTACC GCAGGGCCATATTATGATTCCGGGGATCCGTCGACC	pED208Δ*traE*
AA8_traE_delR	ATTCGTCACCTCATAGAACCGACCGATACGGGTAAAG CCTCCGGTGTACTTCTGTAGGCTGGAGCTGCTTCG	pED208Δ*traE*
AA3_traG_delF	GATGAGCGACCGTGTGCTGGGTAACTACCAGTTCAGC AGGGTGAATTGATATGATTCCGGGGATCCGTCGACC	pED208Δ*traG*
AA4_traG_delR	GTTATCTTTGATATCATTCAATTCCTTTCTCCCTACAC CATTATCACCGATCCTGTAGGCTGGAGCTGCTTCG	pED208Δ*traG*
AA27_traGchk_F	ACATCGCGGTCGACATCTATCTGC	pED208Δ*traG*
AA28_traGchk_R	CGCTTCCTAAAGAAATTTTCAGGC	pED208D*traG*
AA9_traK_delF	CGGAGGCTTTACCCGTATCGGTCGGTTCTATGAGGTG ACGAATGAAAAATAACATTCCGGGGATCCGTCGACC	pED208Δ*OMCC* construction
orb237	CGTTCAGAAATTTTTTCACCCGAAACTCCTTAATTTAC CCTGGCCGGAAGTGTAGGCTGGAGCTGCTTCG	pED208Δ*OMCC* construction
AA31_traKchk_F	CAATGTCTCGCCTGAAACGG	pED208Δ*OMCC* confirmation
orb239	GTCCAGACTGGCCAGATTGC	pED208Δ*OMCC* confirmation
orb212	CCTGATGAGATGCGCGAACTTGAAGCGCTTGCGGAG GCTGCATGACCACTATTCCGGGGATCCGTCGACC	pED208ΔFspecific
orb201	ACGGTGTAAATCGTATCAACGGCCATATCAATTCACC CTGCTGAATGTAGGCTGGAGCTGCTTCG	pED208ΔFspecific
orb214	GACGCTGTATACCAGCTCGC	pED208ΔFspecific
orb207	CACGCCATTGAGCGAATCCT	pED208ΔFspecific
AA19_traLcl_F	AACTAGCTAGC AGGAGG AATTCACCATGGAGGGAAACGATTTAGACAAATAC	pAM3
AA20_traLcl_R	AACCCAAGCTTGCGGTAATTTATTTTATCCATTTCCTG	pAM3
AA21_traEcl_F	AACTAGCTAGCAGGAGGAATTCACCATGGAAATTAG CGCTCGTAATTCAT CCACC	pAM4
AA22_traEcl_R	AACCCAAGCTTTTATTTTTCATTCGTCACCTCATAGAA CC	pAM4
AA17_traG_NH_F	AACTAGCTAGCAGGAGGAATTCACCATGGCCGTTGAT ACGATTTACAC	pAM1
AA18_traG_Hd_R	AACCCAAGCTTCCCTACACCATTATCACCGATCC	pAM1
Del-traB(pOX38)-Frt-Km_F1	CATGGATGTGTATGTCATCCGTGACGGGGAGGGCAAC TGATGATTCCGGGGATCCGTCGACC	FΔ*traB*
Del-traB(pOX38)-Frt-Km_R1	GCCATCGTTGCCCCCTCCCTGGCTGCCGGACATAAAC TGCTGCGGATTCTGTAGGCTGGAGCTGCTTCG	FΔ*traB*
Up-traB-pOX38_F	CGCTGTCTGCCCTGAATATCCGG	FΔ*traB*
Dn-traB-pOX38_R	GGCAGAATGACGGTGTACTTCAGAC	FΔ*traB*
orb278	TTGAAGAAGGATTTTGTGAACTGCGATGAGAAAGGT GAGGGACAATGAAAATTCCGGGGATCCGTCGACC	FΔ*traV*
orb279	TGCCGGCTGATATACAACTTCAGGGCCAGTTAATTAA TACGTGGTTTTCCTGTAGGCTGGAGCTGCTTCG	FΔ*traV*
orb296	GTGCACCAGAACCTGATGCG	FΔ*traV*
orb297	CGGAATATTCCTCCCGACATGTTC	FΔ*traV*
orb335	GACCTGGCTGGATAATTTCGGGGAAACAGACGATGA GAAAAAATAATACGATTCCGGGGATCCGTCGACC	FΔ*traK*
orb336	GCGTTTCACAATGGTATTGATACTGGCCATCAGTTGC CCTCCCCGTCACGTGTAGGCTGGAGCTGCTTCG	FΔ*traK*
orb339	CTGGCCTCAGTATGGCCGTG	FΔ*traK*

orb340	CCGGACATGTCCACATCAGACAG	FΔ*traK*
Del-traL(pOX38)-Frt-Km_F	TGCTGTGGGTATGGCCGTCGTTGGCCTCTGACAGGAA ATAAAACGATGATTCCGGGGATCCGTCGACC	FΔ*traL*
Del-traL(pOX38)-Frt-Km_R	CGTGTTCCATATAACAATCCTGGTATCAGTTCTATTTA ATCCACTGCCGGAATGTAGGCTGGAGCTGCTTCG	FΔ*traL*
Up-traL-pOX38_F	GGCGACCTTCGGTAAGGACTCC	FΔ*traL*
Dn-traL-pOX38_R	GTGCGTTGCTCATTCTGAAGACGG	FΔ*traL*
Del-traE(pOX38)-Frt-Km_F1	GCAGTGGATTAAATAGAACTGATACCAGGATTGTTAT ATGATTCCGGGGATCCGTCGACC	FΔ*traE*
Del-traE(pOX38)-Frt-Km_R1	CTCATCGTCTGTTTCCCCGAAATTATCCAGCCAGGTC ACCCCGTTTTCTGTAGGCTGGAGCTGCTTCG	Fv*traE*
Up-traE-pOX38_F	CGGTTCTGGTTTATTTCGGGATTAAAAAACTG	FΔ*traE*
Dn-traE-pOX38_R	GATTCGGGCTGGTATTGCTGATGGC	FΔ*traE*
Del-traC(pOX38)-Frt-Km_F	CCTTCATGGCTACCGCATTTCTCGCTTTATTTTTCAAC TAAGGAATTCATGTGATTCCGGGGATCCGTCGACC	FΔ*traC*
Del-traC(pOX38)-Frt-Km_R	GCCGTGACGTCGGCGGGTTTCTGCGTTGAACTCATGC CACACTCCTGTATTTTGTAGGCTGGAGCTGCTTCG	FΔ*traC*
up-traC(pOX38)_F	GTCAGTCGCAGAACATAGTGATTTAATTCC	FΔ*traC*
dn-traC(pOX38)_R	GCTTCTGACAGTTGTTTCTGACTGGC	FΔ*traC*
Del-traD(pOX38)-Frt-Km_F1	AATCAGTCCGACTGACTTTTTTTCTTCGGAATATCATC ATGATTCCGGGGATCCGTCGACC	FΔ*traD*
Del-traD(pOX38)-Frt-Km_R1	CCTGTTCATCAGAAATCATCTCCCGGCTCAACATCCT CCCCGCGCTCCCGTGTAGGCTGGAGCTGCTTCG	FΔ*traD*
Up-traD-pOX38_F	CACTGAGCGGTTGTGCAAATTCTTTGTG	FΔ*traD*
Dn-traD-pOX38_R	GGCCATTTGATCAAATGATCGCAGGTC	FΔ*traD*
Del-traG(pOX38)-Frt-Km_F	CTCAGTCGTTACCAGAACAACTATCACTTCGGAGGGA GCACGCTGTGAATATTCCGGGGATCCGTCGACC	FΔ*traG*
Del-traG(pOX38)-Frt-Km_R	CTCTCCATACCCTACCCAACATGTTATGATTATTCTTT ATGCTGGTAACTTGTAGGCTGGAGCTGCTTCG	FΔ*traG*
Up-traG-pOX38_F	GATTGCGGCGTTTCAGTCACAGGTG	FΔ*traG*
Dn-traG-pOX38_R	GGTTTTAATCCCTGCCATAGTGACGG	FΔ*traG*
traB(pOX38)-NheI_F	CTAGCTAGCAGGAGGAATTCACCATGGCCAGTATCAA TACCATTGTGAAACGC	pBAD24-traB(F)-Strep
traB(pOX38)-Strep-HindIII_R	CCCAAGCTTTCACTTTTCGAATTGAGGATGAGACCAT TTGCCATCGTTGCCCCCTCCCTG	pBAD24-traB(F)-Strep
orb360	AGAGAGGCTAGCAGGAGGAATTCACCATGAGAAAAA ATAATACGGCAATAATATTCGGC	pBAD24-traK(F)
orb361	AGAGAGAAGCTTTCAGTTGCCCTCCCCGTCAC	pBAD24-traK(F)
orb294	AGAGAGGCTAGCAGGAGGAATTCACCATGAAACAGA CTTCTTTCTTTATTCCTCTGC	pBAD24-traV(F)
orb295	AGAGAGAAGCTTTTAATTAATACGTGGTTTTCCCCAC GC	pBAD24-traV (F)
KKTX259	AGAGAGGCTAGCAGGAGGAATTCACCATGTCGGGAG ACGAGAATAAACTTAAG	pKKF079
KKTX260	AGAGAGAAGCTTCTATTTAATCCACTGCCGGAAACAC G	pKKF079
KKTX257	AGAGAGGCTAGCAGGAGGAATTCACCATGGAACACG GTGCCCGTTTAAGTAC	pKKF078
KKTX258	AGAGAGAAGCTTTTATTTTTTCTCATCGTCTGTTTCCC C	pKKF078
traC(pOX38)-NheI_F	CTAGCTAGCAGGAGGAATTCACCGTGAATAACCCACT TGAGGCCGTC	pPK22
traC(pOX38)-HindIII_R	CCCAAGCTTTCATGCCACACTCCTGTATTTCTCATG	pPK22
traD(pOX38)-NheI_F	CTAGCTAGCAGGAGGAATTCACCATGAGTTTTAACGC AAAGGATATGACCCAGG	pPK31
traD(pOX38)-HindIII_R	CCCAAGCTTTCAGAAATCATCTCCCGGCTCAACATC	pPK31
traG(pOX38)-NheI_F	CTAGCTAGCAGGAGGAATTCACCGTGAATGAAGTTTA TGTGATTGCCGGTGG	pBAD24-traG(F)-Strep

traG(pOX38)-Strep-HindIII_R	CCCAAGCTTTCACTTTTCGAATTGAGGATGAGACCATTCTTTATGCTGGTAACTCTTTGCTTTCTC	pBAD24-traG(F)-Strep
KKTX584	CTAGCTAGCAGGAGGAATTCACCATGATAAGTAAACGCAGATTC	pKN3
KKTX585	CCCAAGCTTTTACTTTTCGAATTGAGGATGAGACCAGTGAGAGACATGTCCGCCCT	pKN3
KKTX636	TCCATACCCGTTTTTTTGGGCTAGCATGGCCAACGTTAATAAAG	pKN9
KKTX531	GGGCTTTTTAGGCCCGGTATCC	pKN9
KKTX574	CGGGCCTAAAAAGCCCTCACTGCCGTACATTCCGTCAGG	pKN9
KKTX637	CTCATCCGCCAAAACAGCCAAGCTTTTACTTTTCGAATTGAGGATG	pKN9, pKN10. pKN11, pKN12
KKTX638	TCCATACCCGTTTTTTTGGGCTAGCATGGCCAGTATCAATACC	pKN10, pKN11, pKN12
KKTX573	GAAGAAATCCACGGGAAACGTGGTCCCTGTTTTCCCTCATTAC	pKN10
KKTX550	CGTTTCCCGTGGATTTCTTCCGGC	pKN10
KKTX535	GCCCTTGATGCCGTTCTTGCCCATAAAGGAC	pKN10
KKTX567	CGTTACGCATCACCACTTCGCCCTTGATGCCGTTCTTGCCCATAAAGGAC	pKN10
KKTX540	GTTACGCATCACCACTTCGCCC	pKN11
KKTX568	CGAAGTGGTGATGCGTAACGGCCAGATCCTGCTGTATGCAGG	pKN11
KKTX569	TCGGTATCACCGGGTGATATTGTTCAGCACGTTTGATGTAGTAATCA	pKN11
KKTX560	GTGATACCGATTGGTGCGG	pKN11
KKTX570	ACGTGCCGAACAGTATCACCCGGTCATTCCGATTGGCGCAGGTAATG	pKN12
KKTX544	GTGATACTGTTCGGCACGTTTGAT	pKN12
traB-delAP_F	[phos]TACCACCCGGTCATTCCGATTGGCG	pBAD24-B-del AP
traB-delAP_R	[phos]ACGCATAACCACTTCGCCCTTGATGC	pBAD24 -B-del AP
traB-delloop_5xG_F	[phos]GGCGGCGGCAGCTCAGCCGCCAAAACCCTCTCT	pBAD24-B-del AP loop_5xG
traB-delloop_5xG_R	[phos]GCCGCCAATGCCCTTGCCGATACCATCCAGG	pBAD24-B-del AP loop_5xG
KKTX546	CTAGCTAGCAGGAGGAATTCACCATGGCCAACGTTAATAAAGTCG	pYGL683 and pYGL684
KKTX547	CCCAAGCTTTCACTTTTCGAATTGAGGATGAGACCACTGTGTGACGACCTGTCCGG	pYGL683 and pYGL684
NdeI_strep_KpnI_TraD-OX_F	ATATATATCATATGTGGTCTCATCCTCAATTCGAAAAGGGTACCAGTTTTAACGCAAAGGATATGACCCAGGGC	pYGL342
BamHI_TraD-OX_R	ATATATATGGATCCTCAGAAATCATCTCCCGGCTCAACATCC	pYGL342
NdeI_strp_KpnI_TraD-ED_F	ATATATATCATATGTGGTCTCATCCTCAATTCGAAAAGGGTACCAGCCTGAATCCTCGCGACTTAACGCAG	pYGL343
BamHI_TraD-ED_R	ATATATATGGATCCTCAGTATTCCCTCCCGTCATCCATCTC	pYGL343
TraD-OX-dCT15-BamHI_R	ATATATATGGATCCTCAATTAATGTTCACCTCTTCACGACGCTGCATC	$_{strep}traD\Delta C15_{F}$ construction
TraD-ED-dCT15-BamHI_R	ATATATATGGATCCTCAGTTAATGTTGACCTCTTCCCTGCGGG	$_{strep}traD\Delta C15_{ED}$ construction
TraD-OX_CT15-ED_F	[phos]GATGGATGACGGGAGGGAATACTGAGGATCCGCGGATAAATAAGTAACGATCCGG	pYGL351
TraD-OX_CT15-ED_R	[phos]TCATGCGTTTTGTCTGTCGCATGATTAATGTTCACCTCTTCACGACGCTGCATC	pYGL351
TraD-ED_CT15-OX_F	[phos]GTTGAGCCGGGAGATGATTTCTGATGGGATCCGCGGATAAATAAGTAACGATCCGG	pYGL353
TraD-ED_CT15-OX_R	[phos]ATCCTCCCCGCGCTCCCGGTGCACGTTAATGTTGACCTCTTCCCTGCGGG	pYGL353

TraD-dCT166-ED/d148-OX_F	[phos]TGAGGATCCGCGGATAAATAAGTAACGATCC	pYGL491 and pYGL492
TraD-dCT166-ED_R	[phos]AGCCACCTTAGGCATCGAGTCATACTTC	pYGL491
TraD-dCT148-OX_R	[phos]AGCGACCTTCGGTCGTGTCTGATATTTC	pYGL492
NdeI-TraJ_F	ATATATCATATGGACGATAGAGAAAGAGGCTTAGCATTTTTATTTG	pYGL493 and pYGL494
BamHI-strp-TraJ_R	ATATATGGATCCTCACTTTTCGAATTGAGGATGAGACCAGATCTC	pYGL493
KpnI-strp-TraJ_R	ATATATGGTACCCTTTTCGAATTGAGGATGAGACCAGATCTCC	pYGL494
KpnI-TraD-CT166ED_F	ATATATGGTACCGACGCCCTGCTGTTGCGGGATG	pYGL494
NdeI-TraD-CT166ED_R	ATATATCATATGGTACTCCTTATGGCATTATTGATGACTTGTTAATAAC	pYGL494
SpeI-TraJdNT75_F	ATATATACTAGTGATAAATCACGCGTGTTTAAAGGCGAAAGATTC	pYGL528 and pYGL529
NsiI-TraJdNT75_R	ATATATATGCATATGGTACTCCTTATGGCATTATTGATGACTTG	pYGL528 and pYGL529
NsiI-TraD1-134_F	ATATATATGCATAGCCTGAATCCTCGCGACTTAACGC	pYGL528 and pYGL529
SpeI-TraD1-134_R	ATATATACTAGTGAGATACCAGTACACGGCAAATGTGGC	pYGL528 and pYGL529
NheI-oriTKJI_F	ATATATGCTAGCAGGAGGAATTCACCATGGTACCCTCATTTAGAATGATGTAATTTTGATGTATTTCTGATG	pCGR97
HindIII-oriTKJI_R	ATATATAAGCTTCATCAGATTTCATGGCCCCCTTCTTCATG	pCGR97
dTraJ_F	[phos]CCTGGCATTGAACTGAGGGAGATCTG	pYGL490
dTraJ_R	[phos]CAAATAAAAATGCTAAGCCTCTTTCTCTATCGTCC	pYGL490
NotI-pED208-oriT_F	ATATATGCGGCCGCTGAGCGCATTATCACGCCAGGACG	pYGL249
HindIII_pED_oriT_R	ATATATATAAGCTTTTTGGCATTTCAAAAACCTTATTAGAAATTTAAATTTGTCGG	pYGL249
NotI-pOX38-oriT_F	ATATATGCGGCCGCAATCTACCTGCATCAGTCCGCTGCC	pYGL248
HindIII_pOX_oriT_R	ATATATATAAGCTTGCTGATATACAGGTTCACCTTAGCCATTAG	pYGL248
