# Supplementary material for: Chimeric systems composed of swapped Tra subunits between distantly-related F plasmids reveal striking plasticity among type IV secretion machines
Source: PLoS Genet. 2024 Mar 4;20(3):e1011088. doi: 10.1371/journal.pgen.1011088 (PMC10939261; doi:10.1371/journal.pgen.1011088)
Supplement: S4 Table — (PDF) [file pgen.1011088.s008.pdf]

S4 Table. Transfer frequencies and M13KO7 phage susceptibilities of MC4100 hosts with co-resident F and pED208 mutant plasmids.

		pED208								
		ΔA	ΔL	ΔE	ΔG	ΔC	ΔD	ΔK	ΔV	ΔB
		-	-	-	-	-	1.1×10^{1}	7.0×10^{-4}	1.0×10^{-3}	-
	ΔA	-	-	-	-	-	3.6×10^{-3}	1.6×10^{-2}	9.0×10^{-2}	-
		2×10^{-5}	-	-	-	-	6.9×10^{-1}	9.0×10^{-4}	1.0×10^{-3}	-
	ΔL	3×10^{-4}	-	-	-	-	1.9×10^{-2}	4.0×10^{-2}	2.4×10^{-2}	-
		8.8×10^{-5}	-	-	-	-	2.0×10^{-1}	6.7×10^{-5}	1.7×10^{-4}	-
	ΔE	6.5×10^{-5}	-	-	-	-	6.9×10^{-4}	1.6×10^{-3}	6.8×10^{-3}	-
		7.1×10^{-5}	-	-	-	-	3.4×10^{-1}	2.9×10^{-4}	1.2×10^{-4}	-
	ΔG	4.5×10^{-5}	-	-	-	-	1.2×10^{-3}	2.1 10^{-2}	4.4×10^{-3}	-
F		5.4×10^{-5}	-	-	-	-	3.0×10^{-1}	9.0×10^{-5}	2.3×10^{-5}	-
	ΔC	4.9×10^{-5}	-	-	-	-	8.3×10^{-4}	2.8×10^{-3}	6.3×10^{-4}	-
		4.3×10^{-7}	1.3×10^{-6}	7.8×10^{-7}	1.3×10^{-6}	7.0×10^{-5}	-	1.8×10^{-7}	-	2.7×10^{-7}
	ΔD	2.8×10^{-1}	1.6×10^{-1}	1.6×10^{-2}	8.1×10^{-2}	3.0×10^{-2}	-	5.4×10^{-2}	6.3×10^{-2}	1.8×10^{-2}
		3.1×10^{-3}	2.7×10^{-4}	3.1×10^{-3}	1.8×10^{-3}	1.4×10^{-3}	7.9×10^{-2}	-	4.5×10^{-3}	2.1×10^{-3}
	ΔK	3.5×10^{-5}	2.4×10^{-6}	1.7×10^{-8}	6.7×10^{-8}	5.3×10^{-7}	1.1×10^{-4}	3.5×10^{-6}	6.6×10^{-4}	-
		2.6×10^{-1}	1.5×10^{-2}	3.9×10^{-4}	1.4×10^{-3}	1.3×10^{-3}	2.1×10^{-1}	7.5×10^{-4}	-	2.8×10^{-3}
	ΔV	2.2×10^{-3}	4.6×10^{-4}	8.8×10^{-6}	1.3×10^{-5}	1.1×10^{-5}	1.0×10^{-3}	2.9×10^{-4}	-	1.3×10^{-5}
		1.9×10^{-6}	-	-	-	-	1.2×10^{-1}	-	9.5×10^{-8}	-
	ΔB	9.0×10^{-6}	-	-	-	-	2.6×10^{-3}	2.3×10^{-5}	5.6×10^{-5}	-

		pED208								
		ΔA	ΔL	ΔE	ΔG	ΔC	ΔD	ΔK	ΔV	ΔB
		-	-	-	-	-	-	-	-	-
	ΔA	-	-	-	-	-	3.0×10^{-1}	3.7×10^{-4}	1.1×10^{-4}	-
		-	-	-	-	-	-	-	-	-
	ΔL	-	-	-	-	-	3.8×10^{-1}	4.1×10^{-4}	3.6×10^{-4}	-
		-	-	-	-	-	-	-	-	-
	ΔE	-	-	-	-	-	2.2×10^{-2}	1.0×10^{-5}	5.5×10^{-5}	-
		-	-	-	-	-	-	-	-	-
	ΔG	-	-	-	-	-	2.0×10^{-2}	2.1×10^{-5}	6.3×10^{-5}	-
F		-	-	-	-	-	-	-	-	-
	ΔC	-	-	-	-	-	1.1×10^{-2}	2.0×10^{-5}	2.7×10^{-5}	-
		7.2×10^{-2}	5.5×10^{-2}	2.7×10^{-2}	1.2×10^{-2}	9.4×10^{-2}	1.0×10^{-2}	3.1×10^{-2}	7.3×10^{-3}	1.1×10^{-2}
	ΔD	-	-	-	-	-	1.0×10^{-2}	-	7.3×10^{-3}	-
		2.1×10^{-4}	5.6×10^{-6}	1.8×10^{-6}	1.3×10^{-6}	7.1×10^{-6}	-	-	1.1×10^{-6}	2.3×10^{-7}
	ΔK	-	-	-	-	-	1.0×10^{-2}	-	-	-
		5.2×10^{-2}	3.2×10^{-3}	3.8×10^{-3}	5.4×10^{-3}	4.6×10^{-3}	-	6.1×10^{-3}	-	1.7×10^{-3}
	ΔV	-	-	-	-	-	1.6×10^{-2}	-	-	-
		-	-	-	-	-	-	-	-	-
	ΔB	-	-	-	-	-	5.6×10^{-2}	2.7×10^{-7}	2.8×10^{-6}	-

Upper: Matings with donors harboring co-resident F and pED208 mutant plasmids were plated on media selective for transconjugants carrying F or pED208. Transfer frequencies are reported for F (green shading) and pED208 (blue shading) variants. **Lower:** M13KO7 phage susceptibilities of strains harboring co-resident F and pED208 mutant plasmids, reported as the number of kanamycin-resistant (Kan^R) transductants per total colony-forming units. Infection frequencies are color-coded based on the predicted type of pilus (F, green; pED208, blue) the host strain produces, rendering M13KO7 susceptibility. Matings and phage infection assays were repeated at least three times in triplicate; the average frequencies of plasmid transfer (Tcs/D) or phage infection (Kan^R/CFUs) are shown for a representative experiment. “-“, no plasmid transfer or phage susceptibility. These tables present data for images presented in Fig 7B; source data are in S5 Table.
