# Supplementary material for: Chimeric systems composed of swapped Tra subunits between distantly-related F plasmids reveal striking plasticity among type IV secretion machines
Source: PLoS Genet. 2024 Mar 4;20(3):e1011088. doi: 10.1371/journal.pgen.1011088 (PMC10939261; doi:10.1371/journal.pgen.1011088)
Supplement: Attachment — Submitted filename: PLOSGeneticsReviewerResponses(1).docx [file pgen.1011088.s010.docx]

**Reviewer Responses:** We thank the reviewers and editor for positive comments and support of our work. We have addressed all comments in a revised manuscript, as itemized below. We also have included all source data for the conjugation and M13K07 phage assays, and for the bioinformatics analyses presented in Fig. 7A.

**Editor's comments:** 
As you will see in the reviews below, all three reviewers are enthusiastic about the initial submission. But all have highlighted some minor points for you to consider, which should be addressed by changes to the text, additional analysis of existing data, and/or by performing additional experiments as indicated.

**Reviewer Response:** We have incorporated all requested edits and provided additional experimentation to shore up fundamental conclusions. We are currently pursuing two lines of experimentation requested by reviewer 3 and plan on developing the findings into independent publications. As justified further below, we believe these additional investigations are tangential to the present study and beyond the scope of this already large body of work.

**Reviewer #1:** Bacterial conjugation is a paramount research topic due to its significant contribution to the dissemination of antibiotic resistance in bacteria. Autonomous conjugation plasmids encode the machinery enabling their transmission to other bacterial cells, including a conjugative type IV secretion system (T4SS), strictly essential to transport the DNA through the membranes of the donor and recipient cell engaged in the mating process. T4SSs from different conjugation plasmids are composed of various conserved subunits, yet they also exhibit diversity in composition and structure. T4SS gene systems encode a type IV coupling protein (T4CP), an inner membrane complex (IMC), an outer membrane core complex (OMCC), and factors involved in the production of the conjugative pilus. In the manuscript "Chimeric systems composed of swapped Tra subunits between distantly-related F plasmids reveal striking plasticity among type IV secretion machines," Kishida and co-workers report the results of an ambitious study aiming at clarifying how T4SSs recruit specific substrates, elaborate functional channels, and in some cases build the conjugative pili. To do so, they implemented a well-designed genetic dissection involving systematic deletion/complementation and subunit swapping, and they assessed the functionality of the resulting chimeric machines. They proceed by swapping individual Tra/Trb subunits between T4SSs encoded by the phylogenetically distantly related F-like plasmids, F and pED208. The chimeric systems are examined in terms of the ability to produce functional conjugation machinery as addressed by the efficiency of plasmid transfer) and ability to produce the conjugative pilus as reported by fluorescence microscopy imaging and sensitivity to M13 and MS2 phages, which adsorb the tip and to the side of the conjugative pilus, respectively. This study produces a wealth of data that helps understand the plasticity and cross-functionality of these two F-encoded T4SSs. The main insights and conclusions that the authors put forward are - The identification of regions of intrinsic flexibility among the encoded T4SSs - The relaxed specificity between TraD and its substrate, and with the T4SS - the identification of which TraD domains mediates the recruitment of cognate or heterologous DNA substrates. The C15 motifs appear to be the most critical for recruiting cognate F-like plasmids. The transfer was less but still stimulated by the CTDΔC15, and even less by the TraDΔCTD - The unanticipated observation of DNA transfer in the absence of detectable pilus in some strains producing chimeric systems. On this last point, this reviewer thinks that the lack of detectable pili using microscopy and sensitivity to M13 infection does not strictly exclude the possibility that some pili might exist in a form, location, or configuration that would not be accessible to M13 phage or the maleimide conjugates. Therefore, some of the interpretations and conclusions might be moderated accordingly (see comments below) - Strains containing "incomplete" F-like and F plasmids (lacking T4SS components required for their autonomous transfer) do elaborate functional chimeric T4SSs enabling plasmid transfer. This elegant study leads to the conclusion that combinations, interactions, and complementarity between T4SS components from different plasmids likely facilitate the propagation of plasmids that are not self-transmissible per se. Overall, the experimental design and results are sound and of high quality. Conclusions are well supported by the data (though potentially overstated regarding the absence of detectable pili). In summary, this work constitutes a significant addition to the field of conjugation biology and the understanding of the plasticity of type IV secretion machines.
**Reviewer Response:** Thanks very much for the detailed and positive summary of our study. We agree with the reviewer’s comments about the role of pili or at least of pilins in DNA transfer and we have modified the text accordingly, as described further below.

1- Figure 1Bi and ii: This might be due to a misunderstanding of the figure, but this reviewer feels that the transfer value shown for TraDED complementation in panel Bi should be the same as for TraDED complementation in panel Bii (as is the case for panel Ci and Cii).
Also, have the authors tryied to produce the traDEDC15F in the pED208del(traD) and vise versa, i.e., the traDFC15ED in the pFdel(traD)? Would that be an interesting addition to the data set by producing firther insights regardnig the role of the C15?
An additional minor remark is that the individual data points are hardly visible on the blue bars. Though this requires the editing of most figures, the authors could consider using a lighter blue.

**Reviewer Response:** There is some experiment-to-experiment variation in these matings, as evidenced in the matings carried out in Bi and Bii for the deltatraD strain expressing WT traD. We added the requested data for tradEDC15F and traDFC1fED to the figure. The results are compatible with the rest of the analyses, the C15 is highly system-specific and does not elevate transfer through the noncognate T4SS. That is, deletion of the C15 from or fusion of the heterologous C15 to TraD confer similar effects on plasmid transfer. We also lightened up the blue bars in all figures so datapoints are more visible.

2- L191-192: "Interestingly, donors harboring pED208deltraD and producing TraJKM supported transfer of pED208deltraD, albeit at a low frequency of ~10^-7 Tc's/D (Fig. 1D). We appended TraDED's CTD to TraJKM, which elevated pED208 transfer by ~10^1.5-fold.". This reviewer agrees that TraJKM enables low transfer frequency of the pED208ΔtraD plasmid; however, the ~101.5-fold increase with the TraJKMCTDED is less convincing due to the low values and respective standard deviations. A statistical test might be required to ascertain the significance of such subtle differences and strengthen the interpretation.

**Reviewer Response:** We agree, and we now state that this is not a statistically significant difference (Student’s *t* test, *p*> 0.05) in the text.

3- L195-196: The statement "TraJKM efficiently mediates the transfer of poriTKM, a plasmid harboring the pKM101oriT sequence, through the T4SSKM" is supported by reference 37. It would be helpful to the reader to indicate the frequency achieved in this context (TC's/D = 10^0 if I am correct?). In the absence of TraD, the frequency drops to 10^-4. The following statement, L197-198, "…showing that TraJKM functionally interacts with both T4SSs…" might convey that TraJKM interacts equally with both T4SSs, which does not appear to be the case. Therefore, the authors might consider restating this conclusion to notify the difference.

**Reviewer Response:** The 10-4 10-6 frequency of transfer through the F-like T4SSs clearly shows that TraJ functionally engages with the F systems - this is a moderate transfer frequency for plasmid transfer through any T4SS. But the reviewer is correct that TraJ doesn’t support transfer of the poriT substrate through the F systems as efficiently as it does through its cognate T4SSKM; we now state this point directly.

4- L200-202: "TraD's NTD/NBD thus recruits a completely heterologous substrate, whereas the C15 motif blocks this recruitment. Finally, strains co-producing native TraDED or TraDF and TraJKM failed to transfer poriTKM, suggesting that the TraD T4CPs outcompete TraJKM for engagement with the cognate T4SS channels". Do the authors have any interpretation regarding the fact that poriTKM transfer is observed in the presence of TraJKM and TraDdelCTDED but not in the presence of TraJKM and TraDdelCTDF? Does that teach us anything about the differences between F and pED208 systems?

**Reviewer Response:** We suspect the difference might be attributed to the fact that TraDdelCTDF supports transfer of its F plasmid substrate at a higher frequency than TraDdelCTDED supports transfer of its pED208 substrate. TraDdelCTDF thus might more efficiently engage with its substrate and the T4SSF, which accordingly would reduce the number of machines available for coupling with TraJ/poriT. At this time, we don’t know the mechanistic or structural bases for these differences, therefore we present them simply as observations.

5- Figure 2B: This reviewer understands there might be a mistake in the annotation of the bottom six lines in panel B. If this reviewer is correct, these lines should indicate deltraCF and not ED; traCF and not ED; traCED and not FdeltraGF and not ED; traGF and not ED; traGED and not F.

**Reviewer Response:** Yes, very sorry, not sure how this occurred. These details have been corrected.

6- L260-263: "Strains of interest were engineered to produce Cys-derivatized TraAED pilins (TraA.C116), shown previously to support pED208 transfer at WT levels [25]. The Cys118 residues are surface exposed on ED208 pili and accessible to labeling with fluorescent maleimide conjugates, e.g., FM488-mal. We and others also have deployed fluorescently-tagged ssRNA phages such as MS2 to decorate F pili [25, 46, 48]." For the sake of exhaustive reference to previous relevant works, the authors might consider citing the recent PMID:37963249 article, where functional fluorescent labeling of the pilus using maleimide conjugates has been reported for Cys-derivatized TraAED and TraAF pilins.
**Reviewer Response:** This nice work is now mentioned and the reference is included here.

7- The addition of microscopy imaging to visualize the pili is a nice and informative addition to the phage infection test. This strengthens the unexpected observation that some chimeric systems can mediate DNA transfer despite the absence of apparent pili at the surface of the cells, as in the case of strains carrying pED208deltraK or FdeltraK. To this reviewer's knowledge and understanding, this is a novel and important observation that compels us to revise some aspects of our conjugation model. At least, it raises one immediate question. Since all conjugation tests have been performed on solid surfaces, it becomes tempting to assess if those cells can perform conjugation in liquid conditions, where the requirement for pili functions differs. Have the authors performed conjugation tests in liquid conditions? This would undoubtedly deepen the interpretation and impact of these results by providing further clues as to how conjugation might occur in the absence of detectable pili.

**Response:** Remarkably, we still detect transfer by all donors with constructs conferring the Tra+ Pil- “uncoupling” phenotypes in broth matings. We present these data in **S4A Fig** and highlight the findings that the ORC substructure and extracellular F pilius are not required even for plasmid transfer in liquid environments.

8- L265-267: The statement "The deltraK mutant hosts also were resistant to infection by M13K07, further showing that the deltraK machines do not even produce "vestigial" pili with only their tips exposed on the cell surface". The authors may or may not be right in their interpretation. In any case, at this stage, this reviewer feels that the word "showing" is excessive since one cannot exclude the possibility that a vestigial pilus would remain located within the T4SS channel without necessarily exposing any TraA pilin that would be accessible to the M13K107 phage (or to the maleimide conjugate for that matter). Therefore, the authors might consider tempering this interpretation. Along the same line, this reviewer feels that the paragraph's conclusion, "These findings firmly establish that F-like pili are completely dispensable for efficient DNA transfer through F-like channels." should be made more cautious. Indeed, the results show that DNA transfer can occur in the absence of detectable pili (using microscopy and phage infection), but do not strictly exclude the potential existence of pili types/forms that would not be detected with those two tests. It would also be helpful to have a section explaining how the authors reconcile their finding that detectable pili are not required for transfer with the essentiality of TraA for DNA transfer in all tested genetic contexts (in this work and the literature).

**Reviewer Response:** The reviewer is correct, and in the summary to this section we now acknowledge the possibility that the Tra+, Pil- mutant strains might still elaborate a vestigial pilus across the distal part of the cell envelope either without or with a surface exposure. In the latter scenario, the pilus tip might have an altered conformation, which blocks it from serving as a receptor for M13. Importantly, results of our in situ CryoET analyses of F machines firmly established that the central cylinder shown to connect the IMC with the OMCC does not consist of TraA pilin. Therefore, if a vestigial pilus assembles within the cell envelope, it must assemble in the distal portion, possibly docking onto the central cylinder to specifically mediate passage of substrates across the outer membrane.

9- The results reported in Figure 7 are pivotal to the work. In particular, panel 7B presents phage sensitivity and plasmid transfer efficiency of strains carrying co-resident pED208 or F plasmid with individual deletion of tra/trb genes. The results recapitulate and generalize the interpretations reported at the beginning of the article, thus showing the soundness of the entire work. However, this reviewer finds a set of results challenging to integrate and understand regarding FΔtraA). Since we have learned from Figure 6 that FdeltraA is not complemented by TraAED, how can we explain that FdeltraA conjugation deficiency is suppressed by the single deletion of deltraDED from the pED208 plasmid? Does this mean that FdeltraA plasmid transfer is mediated by TraDF through a T4SSF that produces a pilus with TraAED pilin? Can the author clarify this point and confirm or correct this interpretation?
**Reviewer Response:** Fig. 6 shows that FΔtraA is not complemented by TraAED. However, Fig. 1 shows that pED208ΔtraD is complemented by TraDF, and, moreover, that TraDF supports transfer of both F and pED208 at fairly high frequencies (~5x10-3 for pED208 and 5x10-1 for F). Therefore, in a strain carrying FΔtraA and pED208ΔtraD, both plasmids are efficiently transferred through the T4SSED/TraDF chimeric machine. The data support this interpretation, not the notion that TraAED incorporates into the T4SSF.

10- The labels Fig. 5, 6, and 7 are missing at the bottom right corner of the corresponding figures.

**Reviewer Response:** To finalize the figures as .tif files, we had to remove the Fig XX designations from the bottom right.

11- The data of M13 infection tests are presented in the supplementary Figure S3 (except those reported in Figure 4), however, none of the raw results for MS2 phage are presented in the manuscript. Can the authors comment on this point?

**Reviewer Response:** We used a quantitative M13 assay based on M13K07-mediated Kanr transduction, which is afforded by the fact that M13 is not a lytic phage. For the MS2 phage assay, we used a simple spot assay, which for this lytic phage suffices to indicate phage susceptibility or resistance.

**Reviewer #2:** The manuscript by Kishida et al. investigates conjugation systems encoded by two distantly related IncF plasmids, pED208 and F, by systematically deleting components or generating chimeric systems with swapped Tra subunits. The experiments reveal domains of the TraD coupling proteins that contribute to recruitment of cognate or heterologous DNA substrates. Many of the chimeric T4SSs retained the capacity to transfer DNA but exhibited defects in pilus production. Overall, the manuscript is well-written and provides important new insights.

Comments:
1. Lines 82-89 describe structural features of the T4SS encoded by the conjugative plasmid R388, and line 93 describes a previous structural analysis of the OMCC encoded by the IncFV plasmid pED208. It would be helpful to clarify in the Introduction that the R388-encoded T4SS is classified as a prototype or minimized system, whereas the pED208-encoded system is classified as an expanded system.

**Reviewer Response:** Done.

2. Line 93 cites a previous structural analysis of the OMCC encoded by pED208 (reference 15, Liu et al. 2022). At least two additional papers have analyzed structural features of the T4SS encoded by pED208 (Amin et al., 2022, reference 14; and a cryo-ET study by Hu et al., PNAS 2019). These papers should also be cited on page 4. It would be helpful to comment in the Introduction whether structural analyses have previously been undertaken for both of the T4SSs analyzed in the current study (pED208 and F) or only the T4SS encoded by pED208.

**Reviewer Response:** All 3 papers are now cited here. Good point regarding the structural analyses, which thus far have been carried out only for pED208). This is now mentioned.

3. Figure S1 provides valuable information comparing genetic features of the two conjugation systems analyzed in the current study. Fig. S1A illustrates that the Tra/Trb genes in these systems exhibit high levels of sequence divergence. To further orient readers, it would be helpful to briefly comment on the corresponding levels of sequence divergence when comparing components of prototype/minimized T4SSs (such as the R388-encoded system) with components of the conjugation systems analyzed in the current study. For example, are components of the two conjugation systems analyzed in the current study more closely related to each other than to components of prototype/minimized T4SSs such as the R388-encoded system?

**Reviewer Response:** Most F and pKM101 (closely similar to R388) homologs are ~18-20 % identical; the outliers are TraV and TraG homologs (6 and 8 %) and TraC homologs (24 %). We mention this lower relatedness between the F/pED208 (F-like, expanded) and pKM101 (minimized) system in the S1 Fig legend.

4. Figure 1A shows components of chimeric systems in solid colors (green or blue) corresponding to their origin, whereas Figure 2 and several other figures show substituted components in blue/green checkered patterns. Suggest adding a brief description to the figure legends to help readers understand the difference between the solid and checkered color schemes.
**Reviewer Response:** Good suggestion, this is now clarified in the figure legends. The color-coding matches the expressed gene/truncation (pED208 - blue; F – green). The checkered bars denote a chimeric protein composed of domains from pED208 and F (or pKM101 TraJ as yellow).

5. Fig. 6C shows one band labeled as TraA-ED, as well as two other bands. Are the two unlabeled bands proteins that cross-react with the antiserum? Consider commenting on this in the figure legend.

**Reviewer Response:** These are crossreactive species, which is now mentioned in the figure legend (The traA-ED AB is polyclonal, whereas that to TraAF is monoclonal, hence, the blot is cleaner).

**Reviewer #3:** This exceptional manuscript by Kishida et al. describes the generation and analysis of chimeric conjugative type IV secretion systems (T4SS) used to decipher the role of multiple components in apparatus biogenesis and interbacterial DNA translocation. Using interchangeable pED208 and F pilus constituents, the authors thoroughly investigated the mechanistic details underscoring machinery assembly, DNA substrate recruitment, secretion channel integrity, and conjugative pilus biogenesis. This work exploits well-controlled and extensive mutagenesis studies and systematic functional analyses to dissect protein architectures and domains that govern substrate recruitment and translocation channel assembly among artificial and endogenous T4SS chimeras. Notably, the authors convincingly demonstrate subunit interchangeability among isolates harboring non-selftransmissible co-resident T4SS variants, highlighting the clinical and evolutionary importance of these findings within the context of functional diversification and apparatus structural flexibility. Overall, the experimental approaches are rigorous, the manuscript is well written and logically presented, and the findings represent an exciting and novel advance in the T4SS field. In the interest of strengthening the conclusions, the authors should address the following minor comments:

1. In line 162, the authors speculate that truncation of TraD (lacking the C-terminal discrimination motif) attenuates translocation of pED208 and F through the cognate T4SS machineries due to loss of TraD-TraM interactions. Can this hypothesis be tested using bacterial two-hybrid or co-immunopurification approaches?

**Reviewer Response:** Excellent suggestion and we are currently developing another manuscript aimed at further defining the TraD – relaxosome contacts using these and other approaches. As the TraD – TraM interaction has been structurally solved by Glover et al., we feel justified in suggesting that the C-terminal truncation abolishes the TraD – TraM contact; our mating data are also consistent with those reported by Glover et al. in their structural study. We feel that a further comprehensive definition of the TraD – relaxosome contacts is beyond the scope of this already large study.

2. In experiments designed to analyze the contribution of TraB AP on DNA conjugation (lines 321 – 327), several pilus uncoupling mutations were identified that enabled substrate transfer in the absence of vestigial or extracellular pilus assembly. In addition to testing AP variants in which residues are substituted with glycine (e.g., TraBAPL5GED), the authors should analyze whether increased or decreased AP length or loop rigidity influences substrate transfer or pilus biogenesis. While the AP is required for conjugation (Fig. 4), does the AP length or local flexibility alter conjugation frequencies, translocation channel formation, or pilus assembly?

**Reviewer Response:** The reviewer requests some additional mutations along the lines of a study carried out by T. Cover and his colleagues on the Cag system. In that study, however, the CagY AP loop was substituted by an equivalent length GS tract, and then truncations of that tract were phenotypically characterized. Here, we simply deleted the entire TraB AP loop leaving only a 5xG tract so that the two alpha helices of the AP could assemble properly in the OM. The TraB 5xG variant supported efficient plasmid transfer (10-2 Tc’s/D) compared with complementation with WT TraB (10-1 Tc’s/D), establishing that the entire loop is dispensable for efficient plasmid transfer. Consequently, analysis of TraB AP variants with different loop lengths is unlikely to impact – positively or negatively – plasmid transfer. Now, the 5xG mutation does nearly abolish pilus production, so further analyses of AP truncations might show a correlation between the AP loop length or specific sequences within the AP loop and piliation. We are looking into this possibility, but our finding that the 5xG mutation confers a Tra+, Pil- phenotype constitutes the major finding of importance for the present study, namely, that the loop is required for piliation but not substrate transfer, e.g., establishment of the mating junction. As with TraD (see above comment ) as well as nearly all of the other Tra/Trb subunits analyzed in this study, important follow-up work with analyses of additional mutants is needed for refined mechanistic insights; this will provide the datasets for several follow-up papers.

3. In lines 535 – 536, the authors propose that co-resident TraV or TraK are more readily incorporated into heterologous T4SS machineries after the subunits first adopt the correct folded structures within the cognate apparatus. In this scenario, do the authors suggest that OMC subunits disassociate/disassemble and re-assemble into adjacent or nearby heterologous systems? Could disassembled subunits remain in the correctly folded conformation in the periplasm/outer membrane and stochastically incorporate into nascent heterologous subcomplexes/subassemblies during apparatus biogenesis? This speculative model should be more completely elaborated.

**Reviewer Response:** Some of the data that we present suggests this, but we additionally discovered that strains carrying pED208DeltaTraK or pED208DeltaTraV and engineered to produce just the F-encoded OMCC (TraB/TraV/TraK) transfer the plasmid and also elaborate pili at considerably higher frequencies than the strains that bear these plasmids but lack the F-encoded OMCC. We know that TraB is not exchangeable, most likely because its N-terminal region forms specific contacts with the cognate IMC components. So, the most reasonable explanation is that the assembled OMCC facilitates folding of TraV and TraK, which in turn facilitates their subsequent integration into the pED208 machine to a greater extent than if TraV or TraK are produced in the absence of the rest of the OMCC. An alternative possibility is that the F-encoded OMCC somehow functionally coordinates with the pED208 machine without substituting its TraV or TraK components, but how this could occur is a mystery. At this point, while this interesting observation clearly warrants further study, here we simply suggest possibilities to explain the data. We elaborated a bit in the text to clarify our current thinking. For example, because of TraV’s highly unusual structure in the assembled ORC belt, it seems reasonable that preassembly of TraV, or at least assembly of the dimeric structural unit, within the context of the F OMCC could predispose TraV dimers or the intact belt for integration into the pED208 system.

4. In light of two very recent papers describing ssDNA transfer through the F pilus (PMID: 37963249) and pED208 (PMID: 38051116), the results of this study represent a significant advance supporting a model of high efficiency DNA conjugation in the absence of observable pilus biogenesis. Given the results of the current study, can the authors provide additional comment/speculation regarding the role of pili versus proximal mating junctions in substrate delivery? Perhaps the major role of pilins is to stabilize the secretion channel during substrate transfer (while extracellular pili mediate low efficiency DNA translocation)?

**Reviewer Response:** We expanded on this a bit. Clearly, elongated extracellular pili are not required for efficient F plasmid transfer. From a structural perspective, however, it remains possible that the Tra+, Pil- mutants still possess a vestigial pilus that extends through a portion of the cell-envelope-spanning translocation channel. In the absence of high-resolution structures, we can still only speculate how TraA pilins, vestigial pili or even the F pilus contribute to DNA transfer.
